# Oligodendrocyte Progenitor Cell Transplantation Reduces White Matter Injury in a Fetal Goat Model

**DOI:** 10.1111/cns.70178

**Published:** 2024-12-17

**Authors:** Yan Yue, Bixin Deng, Yan Zeng, Wenxing Li, Xia Qiu, Peng Hu, LiuHong Shen, Tiechao Ruan, Ruixi Zhou, Shiping Li, Junjie Ying, Tao Xiong, Yi Qu, Zuo Luan, Dezhi Mu

**Affiliations:** ^1^ Key Laboratory of Birth Defects and Related Diseases of Women and Children (Sichuan University), Ministry of Education, NHC Key Laboratory of Chronobiology Sichuan University Chengdu China; ^2^ College of Veterinary Medicine, Sichuan Agricultural University Chengdu China; ^3^ Laboratory of Pediatrics The Sixth Medical Center of PLA General Hospital Beijing China

**Keywords:** cell transplantation, hypoxic ischemia, oligodendrocyte progenitor cells, preterm white matter injury, umbilical cord occlusion

## Abstract

**Background:**

Preterm white matter injury (PWMI) is the most common type of brain injury in preterm infants, in which, oligodendrocyte progenitor cells (OPCs) are predominantly damaged. In this study, human OPCs (hOPCs) were administered to a fetal goat model of PWMI to examine the differentiation potential and therapeutic effects of the cells on PWMI.

**Methods:**

Preterm goat fetuses were subjected to hypoxic‐ischemia (HI) via intermittent umbilical cord occlusion (5 min × 5). Twenty million hOPCs were administered via a nasal catheter 12 h after an HI insult, and brain tissues were collected 14 or 21 days after the HI insult. Myelin basic protein (MBP) and myelin‐associated glycoprotein (MAG) were detected by immunofluorescence and western blotting techniques. The percentage of myelinated nerve fibers and g‐ratio were examined using transmission electron microscopy. Inflammatory cells were detected by immunohistochemistry. Inflammatory and neurotrophic factors were measured using enzyme‐linked immunosorbent assay.

**Results:**

Our results showed that intermittent umbilical cord occlusion induced PWMI in fetal goats. Transplanted hOPCs can survive in periventricular and subcortical white matter. Further, transplanted hOPCs expressed markers of mature oligodendrocytes (MBP and MAG) and few cells expressed markers of preoligodendrocytes (NG2 and A2B5), suggesting that these cells can differentiate into mature oligodendrocytes in the brain. In addition, hOPCs administration increased MBP and MAG levels, percentage of myelinated nerve fibers, and thickness of the myelin sheath, indicating a reduction in PWMI. Furthermore, hOPCs did not increase the inflammatory response after HI. Interestingly, hOPC administration decreased tumor necrosis factor‐alpha and increased glial‐derived neurotrophic factor and brain‐derived neurotrophic factor levels after HI, suggesting that additional mechanisms mediate the inflammatory microenvironment and neuroprotective effects.

**Conclusions:**

Exogenous hOPCs can differentiate into mature oligodendrocytes in fetal goats and alleviate HI‐induced PWMI. Transplantation of hOPCs is a promising strategy for treating PWMI.

## Graphical abstract

Preterm white matter injury (PWMI) is the most common type of brain injury in preterm infants. In this study, human OPCs (hOPCs) were administered to a fetal goat model of PWMI. Exogenous hOPCs can differentiate into mature oligodendrocytes in fetal goats and alleviate hypoxia‐ischemia‐induced PWMI.

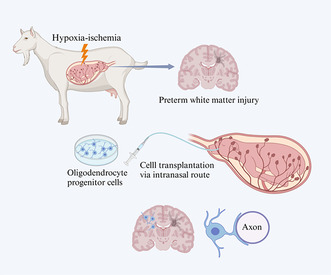

## 1 Introduction

Preterm birth is challenging for neonatal healthcare [1]. Although the survival rate of preterm infants increases with advancements in neonatal intensive care; the incidence of clinical complications including brain injury, is still high [2]. Brain injury in preterm infants is predominantly caused by hypoxic ischemia (HI) or infection [3, 4, 5]. White matter injury is the most common type of brain damage in preterm infants, leading to impaired cognition, decreased physical functioning, and psychological disturbances [6]. Currently, no clinical treatment options are available for preterm white matter injury (PWMI). The main pathological change in PWMI is a disturbance of myelination progression caused by abnormal differentiation and maturation of oligodendrocyte precursor cells (OPCs) [7]. OPCs transplantation is a promising treatment option for PWMI, as the transplanted cells can replace damaged cells in the white matter [8, 9, 10]. Some studies have reported that OPC administration has therapeutic benefits in animal models of leukodystrophy and spinal cord injury [8, 11]. In this study, we investigated the effects of OPCs administration on PWMI.

Animal models for PWMI is a prerequisite for future studies. Most previous studies have used rodent PWMI models. However, as rodents are nongyri with low white matter content, WMI induced in rodents is structurally and physiologically different from that of humans [12]. Goats and sheep are multigyri animals with abundant white matter and a brain structure similar to that of humans [12, 13]. In addition, myelination begins after birth in rodents, whereas myelination begins during mid‐late pregnancy in goats and sheep (similar to humans) [12]. Further, the weight of newborn goats or sheep is also similar to that of newborn humans. Therefore, simulating human PWMI in fetal goats and sheep is feasible. The establishment of fetal sheep models of PWMI has been previously reported [14, 15, 16, 17]. As sheep were unavailable in our area, we employed a goat PWMI model in this study. In this study, global HI was induced by umbilical cord occlusion (UCO) in fetal goats at 100–105 days (term 145 days) of gestational age, a period similar to 24–28 weeks of gestational age in humans [12, 18], to mimic the pathogenic damage caused by PWMI [15, 19].

Choosing a suitable OPCs transplantation pathway is critical for ensuring the curative effect of cell therapy. The commonly used transplantation pathways for cell administration such as systemic administration and direct injection, have some unavoidable disadvantages. For instance, the blood–brain barrier makes it difficult for donor cells to enter the central nervous system after systemic administration [20]. However, direct injections may result in brain injury [21]. Therefore, these strategies are not feasible for clinical applications. The development of an alternative strategy for cell administration is necessary for the treatment of neurological disorders. Previous studies have showed that intranasal administration to be a promising therapeutic approach. Peptides, drugs, viruses, bacteriophages, and cells can enter the brain via the olfactory nerve, trigeminal nerve, blood, cerebrospinal fluid, and lymphatic pathways after intranasal administration [21, 22, 23, 24, 25], demonstrating that intranasal administration is a feasible route for OPCs administration. Given the efficiency of human OPCs (hOPCs) to bypass the blood–brain barrier without the need of invasive procedures, we administered hOPCs intranasally.

In the present study, a PWMI model was established in fetal goats. The efficacy and safety of hOPC transplantation has also been investigated in large animal models. Additionally, we established a nasal delivery route for donor cells in a fetal goat PWMI model. The feasibility of nasal transplantation of hOPCs was also explored. This study provided theoretical and technical support for the clinical research on hOPC transplantation.

## 2 Materials and Methods

### 2.1 Animals and Surgical Preparation

The study design and experimental protocol were in accordance with the institutional guidelines for animal experiments and approved by the Research Animal Care Committee of Sichuan University. Female goats weighing 55–65 kg bearing twin fetuses with a gestational age of 100–105 days (term, approximately 145 days) were used. All animals were purchased from the Chengdu Golden Goat Technology Company, and mating dates were recorded to accurately calculate the gestational age. Ultrasonography was performed to determine pregnancy status and the number of fetuses. Female goats were transported from the farm to the experimental center 2 days before surgery. The goats were maintained in cages and were fasted for 12 h prior to surgery. Surgery was performed in a clean operating room at the Animal Hospital of Sichuan Agricultural University.

### 2.2 Experimental Design

**FIGURE 1:**
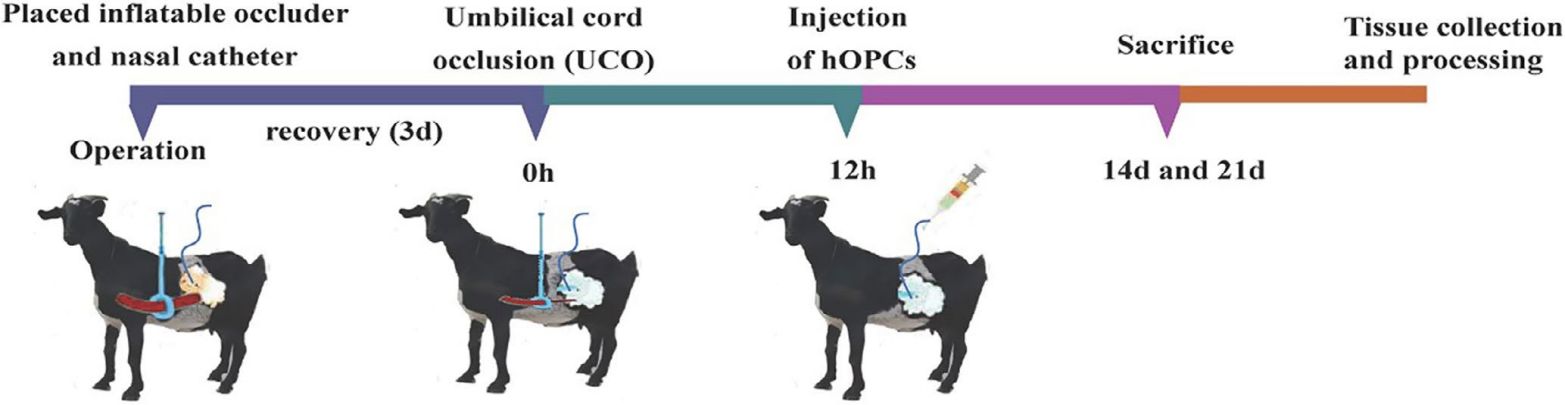
Workflow of preterm white matter injury model establishment and human oligodendrocyte precursor cells (hOPCs) administration. The female goat and fetus underwent surgery to insert an inflatable occluder and nasal catheter. After 3 days of recovery, umbilical cord occlusion (UCO) was performed on the fetus to induce hypoxic ischemia (HI). Twelve hours after UCO, hOPCs were injected through the nasal catheter. The fetus was sacrificed at Days 14 or 21 after UCO.

The general workflow of the PWMI model and hOPCs administration is shown in Figure 1.

Thirty‐six surviving fetuses were used for this experimental study. Each fetus was assigned to one of three groups: (1) HI + OPCs (*n* = 12; 6 fetuses were sacrificed at Day 14 after HI; 6 fetuses at Day 21 after HI); (2) HI + NS (normal saline) (*n* = 12; 6 fetuses were sacrificed at Day 14; 6 fetuses at Day 21); or (3) controls (twin siblings of groups 1 and 2) (*n* = 12; 6 fetuses at Day 14; 6 fetuses at Day 21).

Intravenous access was established at mother's forelegs and propofol (0.5–1 mL/kg) was infused to induce anesthesia. Surgery was performed under general isoflurane anesthesia. The depth of anesthesia and maternal vital signs were constantly monitored by the anesthetic staff. Electrocardiogram monitoring was performed during surgery, and saline was infused intravenously at a rate of 150–250 mL/h. To establish the HI model, the umbilical cord was wrapped in an inflatable silicone occluder (VO‐16; Holly Specialty Products). Saline was temporarily perfused into the occluder from the tail to inflate it, and its ability to obstruct blood flow in the umbilical cord was validated. A sterile nasal polyvinyl catheter was inserted into one side of the nasal cavity to reach the cribriform plate and establish the administration route. Length of the inserted catheter measured approximately the length of the distance from the midpoint of the eyes to the tip of the nose (the anatomical distance was obtained via measurements using fetal goat carcasses at the same gestational age). Both the nares were sealed using sutures. The tail of the occluder and nasal catheter were exteriorized through an incision in the flank of the mother. The remaining twins in (control group) did not receive occluders or nasal catheters. The primary surgical procedure is illustrated in Figure S1. Postoperatively, the mothers were administered antibiotics (ceftiofur sodium, 1 g/d, intramuscular injection) for 3 consecutive days. All the wounds were disinfected daily. The incision condition, mental state, diet, and exercise of pregnant mothers were observed and recorded daily.

After 3 days of recovery, HI was achieved by intermittent clamping of the umbilical cord of the fetus by inflating the occluder under heart rate monitoring using ultrasound. Intermittent clamping involved clamping for 5 min and releasing for 1 min over five cycles. If the fetal heart rate decreased to 60 beats per minute (bpm), clamping was discontinued. Subsequent clamping was performed when the fetal heart rate returned to normal. The investigator performing UCO was blinded to the allocation.

### 2.3 Cell Preparation and Administration

The hOPCs were prepared and provided by the PLA General Hospital, Beijing, China, according to the guidelines approved by the Institutional Review Board of the Hospital. The hOPCs were differentiated from human neural stem cells. Briefly, human neural stem cells were cultured to form neurospheres. The neurospheres were then dissociated into single cells and cultured in a differentiation medium to generate hOPCs. Cells were placed in a 5% carbon dioxide incubator at 37°C. The medium was changed every 3 days. The hOPCs were passaged at 80% confluency [26, 27]. Identification of hOPCs and the differentiation into mature oligodendrocytes in vitro has been described in a previous study [26]. The hOPCs were cryopreserved using 10% dimethyl sulfoxide in fetal bovine serum. Before transplantation, frozen hOPCs were rapidly thawed in a 37°C water bath and plated in a six‐well plate. Half of the hOPC medium was replaced every 3 days. The hOPCs were passaged at 80% confluency and were harvested after two passages. The viability of harvested hOPCs was approximately 85%–90%. Before administration, hOPCs were labeled with CellTracker CM‐DiI (Invitrogen, UK), a red fluorescent dye, to track the cells in vivo [28]. Dulbecco's phosphate‐buffered saline (D‐PBS) containing 2 mg/L CM‐Dil was used to resuspend the harvested hOPCs. The hOPCs were then incubated in the working solution for 5 min at 37°C and 15 min at 4°C. The labeled hOPCs were washed twice with sterile PBS. Viable CM‐DiI labeled hOPCs (2 × 10^7^ cells per animal) were suspended in 2 mL of sterile saline for intranasal administration 12 h after HI. Thirty minutes before cell delivery, the fetuses received hyaluronidase through a nasal catheter to increase nasal mucosal permeability. Labeled hOPCs were injected using a 5 mL syringe through the tail of the nasal catheter, which was exteriorized at the flank of the mother. The hOPCs were delivered at a constant rate, with a total injection time of 3 min. The HI + NS group received an injection of 2 mL of sterile saline via the nasal catheter.

### 2.4 Tissue Collection and Processing

As reported previously, when hOPCs were exposed to differentiation medium in vitro for at least 7 days, most hOPCs differentiated into oligodendrocytes [27]. Moreover, substantial myelination was not observed in the fetal goat brain until 110–115 days of gestation. Therefore, we collected brain tissue at Days 14 and 21 after HI by anesthetizing the mothers and performing a cesarean section. Blood samples were collected from the umbilical veins of fetal goats and delivered to the laboratory for further cellular and biochemical analyses. The fetuses were removed and euthanized using an intravenous overdose of pentobarbital, and the body and organ weights were recorded. Fetal brains were immediately removed and weighed. Right side of the brain was fixed in 4% paraformaldehyde at 4°C for 5 days. Subsequently, the right side was cut into 5 mm coronal slices and further fixed in 4% paraformaldehyde for an additional 5 days. Tissue slices were embedded in paraffin. Five discontinuous coronal slices per animal were cut at the level of the subventricular zone and stained with hematoxylin and eosin (H&E) to assess pathological changes in the white matter. The regions of interest in the fetal brain included the periventricular white matter (PVWM) and subcortical white matter (SCWM). White matter tissue from PVWM and SCWM of the left side was stored at −80°C for subsequent western blot analysis.

### 2.5 Detection of hOPCs in the Brain

For fluorescence analysis, the fixed tissue was dehydrated using 30% sucrose in PBS for 48 h before embedding in tissue freezing medium. Coronal frozen sections of 10 μm thickness were thaw‐mounted onto slides. The slides were stored at 80°C for future use. The frozen slices were thawed and rinsed twice with PBS. Coronal slices were stained with 40,6‐diamidino‐2‐phenyl‐indole (1:500; Beyotime) for 5 min for nuclear staining. The slices were then sealed and rinsed three times with PBS. Finally, the slices were observed under a confocal laser scanning microscope (Olympus, Tokyo, Japan) to identify CM‐DiI labeled hOPCs.

### 2.6 Immunofluorescence Staining

Coronal sections were rinsed with PBS and permeabilized with 0.3% Triton X‐100 for 15 min. Sections were then immersed in blocking reagent for 30 min and incubated with diluted primary antibody for 12–16 h at 4°C. Myelin basic protein (MBP) (1:500; Abcam, ab62631) and myelin‐associated glycoprotein (MAG) (1:500; Cell Signaling Technology, 9043 s) antibodies were used to detect mature myelin sheaths. Antichondroitin sulfate proteoglycan antibodies (NG2) (1:25; Millipore, MAB2029) and A2B5 (1:100; Millipore, MAB312) were used to identify preoligodendrocytes. Glial fibrillary acidic protein (GFAP) (1:500; Abcam, ab116010) and ionized calcium‐binding adaptor molecule‐1 (Iba‐1) (1:500; Abcam, ab178846) were used to detect astrocytes and microglia, respectively. Furthermore, Ki67 (1:500, Abcam, ab15580) antibody was used to visualize proliferating cells. After rinsing with PBS, the sections were incubated with secondary antibodies (Alexa488 antimouse/rabbit immunoglobulin (Ig) G (1:500; Jackson ImmunoResearch, West Grove, PA, USA)) for 2 h at room temperature. Nuclei were stained with 40,6‐diamidino‐2‐phenyl‐indole (1:500; Beyotime) for 5 min. Fluorescence analysis was conducted using ImageJ software (NIH). An investigator blinded to the experimental groups obtained three sections per animal and captured five random fields of view from the regions of interest in each section. The outcomes were assessed by averaging all fields of view for each animal and all animals in each group.

### 2.7 Immunohistochemistry

Paraffin‐embedded samples were sliced into sections (4–6 μm thick). The paraffin sections were deparaffinized and hydrated. Sections were treated with 0.3% hydrogen peroxide for 30 min at room temperature, followed by washing with ultrapure water. Prior to incubation with the primary antibody, the sections were immersed in citrate buffer in a microwave oven and cooled to room temperature. After washing with PBS, the sections were treated with a blocking solution at 37°C for 30 min. Sections were stained with the following antibodies overnight at 4°C: Iba‐1 (1:500, Abcam, ab178846), CD4 (1:200, Abcam, ab288724), and CD8 (1:200, Abcam, ab237709). The blank control was treated with PBS instead of primary antibody. After washing with PBS, sections were incubated with biotinylated antirabbit IgG for 2 h, followed by washing with PBS. The sections were visualized by incubation with an avidin‐biotin complex kit in conjunction with diaminobenzidine. No staining was observed when the primary antibodies were omitted. For quantification, three sections per animal and three fields of view from the regions of interest were obtained at ×20 magnification. The images were processed using ImageJ software and assessed by an investigator blinded to the experiment. Immunohistochemical outcomes were assessed by averaging all fields of view for each animal and for all animals in each group.

### 2.8 Western Blotting

White matter was homogenized and centrifuged. The supernatant was mixed with loading buffer (Beyotime, Shanghai, China). Biotinylated protein precipitates were separated by sodium dodecyl sulfate‐polyacrylamide gel electrophoresis and transferred onto membranes (Millipore, Billerica, MA, USA). The membranes were then probed with primary antibodies against MBP (1:3000, Abcam, ab62631), MAG (1:3000, Cell Signaling Technology; 9043 s) and mouse anti‐β‐tubulin (1:5000, ZSGB‐BIO, Beijing, China), followed by incubation with peroxidase‐conjugated goat anti‐mouse and anti‐rabbit IgG (1:5000; ZSGB‐BIO) for 1 h. Immunoreactive bands were visualized by electrochemiluminescence (Millipore) and imaged using a ChemiDocTM MP imaging system (Bio‐Rad, Hercules, CA, USA). Quantification was performed using at least six independent samples. Each experiment was repeated three times.

### 2.9 Determination of Inflammatory and Neurotrophic Factors

Concentrations of inflammatory factors—tumor necrosis factor‐alpha (TNF‐α), interleukin‐1 beta (IL‐1β), interleukin 6 (IL‐6), interferon‐gamma (INF‐γ), and neurotrophic factors—glial‐derived neurotrophic factor (GDNF), brain‐derived neurotrophic factor (BDNF), ciliary neurotrophic factor (CNTF), nerve growth factor (NGF), neurotrophin‐3 (NT‐3), neurotrophin‐4 (NT‐4), and neurotrophin‐5 (NT‐5) in white matter were determined using commercially available goat enzyme‐linked immunosorbent assay (ELISA) kits (TNF‐α: Cat# RX1100349G, IL‐1β: Cat# RX1100289G, IL‐6: Cat# RX1100389G, INF‐γ: Cat# RX1100395G, GDNF: Cat# RX2D755866, BDNF: Cat# RX2D2018866, CNTF: Cat# RX2D2018886, NGF: Cat# RX2D2018856, NT‐3: Cat# RX2D2018876, NT‐4: Cat# RX2D755326, NT‐5: Cat# RX2D2018896, Ruixin Biotech, China). All procedures were performed according to the manufacturers' instructions.

### 2.10 Transmission Electron Microscope (TEM)

Approximately 1 mm [3] tissue from the PVWM and SCWM were collected and fixed in 2% glutaraldehyde. The tissues were embedded in epoxy resin and sectioned (0.12 mm). The sections were stained with 1% uranyl acetate. Ultrastructure of the myelin sheath was observed at ×1.0 k magnification using TEM (Hitachi, Tokyo, Japan). Five random fields of view were used for each sample. The observers were blinded to the experimental groups. The percentage of myelinated nerve fibers (MNFs) in each field were determined. The outcomes were assessed by averaging the percentage of MNFs in each field. Thickness of the myelin sheath was analyzed by calculating the g‐ratio, which was defined as the ratio of the inner to outer diameter of the myelin sheath. Briefly, images of myelinated axons were imported to the ImageJ software to measure the inner (a) and outer (b) diameters of the myelin sheaths (g‐ratio = a/b). A small g‐ratio indicated a thick myelin sheath. A total of 150 axons were randomly selected and measured from five fields in each sample by the observers.

### 2.11 Data Analysis

All fetuses that completed the procedure within the sampling timeframe were included in the data analysis. Assessments were performed using coded images or samples. The examiners were blinded to the experimental group. Data are presented as mean ± standard deviation (SD). Statistical analyses were performed using GraphPad Prism 8 software (GraphPad Software, San Diego, CA, USA). For two‐group comparisons, the Kolmogorov–Smirnov test was used to test normality of the data. Student's t‐test was used for data that showed normal distribution and homogeneity of variance. The Mann–Whitney U test was used for data that were not normally distributed. For normally distributed data that did not meet homogeneity of variance, an unpaired t‐test with Welch's correction was used. For three‐group comparisons, one‐way analysis of variance (ANOVA) followed by Tukey's post hoc test was used for data that showed homogeneity of variance. Brown–Forsythe and Welch ANOVA were used for data that did not show homogeneity of variance. Differences were considered statistically significant at *p* < 0.05.

## 3 Result

### 3.1 Body and Organ Weights of Fetuses Among Groups

All fetuses that underwent UCO demonstrated amniotic fluid meconia. The body, brain, and organ weights of all fetuses were measured at the time of sampling (Table 1). At Day 14 after UCO, no statistically significant differences were identified in the body and organ weights among the groups. Conversely, at Day 21 after UCO, a significant difference was observed in body weight among groups (control 2.30 ± 0.12 kg; HI + NS 1.97 ± 0.12 kg; HI + hOPCs 2.04 ± 0.10 kg; *p* < 0.0001). Specifically, both the HI + NS and HI + hOPCs groups had lower body weights than the control group (*p* < 0.0001 and *p* = 0.0005, respectively). However, no significant difference was detected between the HI + NS and HI + hOPC groups (*p* = 0.54). At Day 21, the HI + NS and HI + hOPCs groups exhibited lower brain and lung weights than the control group (all *p* < 0.05). Additionally, the HI + hOPCs group had a higher brain weight (*p* = 0.044) than the HI + NS group, with no significant differences in lung weight.

**TABLE 1:** Comparison of body, brain, and organ weights among groups.

Mean ± SD	14 days				21 days			
	Control (*n* = 6, male = 4)	HI + NS (*n* = 6, male = 3)	HI + hOPCs (*n* = 6, male = 3)	*p*	Control (*n* = 6, male = 3)	HI + NS (*n* = 6, male = 4)	HI + hOPCs (*n* = 6, male = 4)	*p*
Body weight (kg)	1.29 ± 0.26	1.20 ± 0.20	1.20 ± 0.14	0.71	2.30 ± 0.12	1.97 ± 0.12	2.04 ± 0.10	< 0.0001
Brain (g)	30.61 ± 2.39	28.11 ± 3.15	27.05 ± 3.37	0.14	41.61 ± 1.57	37.70 ± 1.18	39.63 ± 0.93	0.0003
Heart (g)	9.15 ± 1.03	10.07 ± 0.53	9.71 ± 0.54	0.13	18.01 ± 2.96	16.98 ± 2.47	16.92 ± 1.34	0.68
Liver (g)	63.60 ± 13.01	58.12 ± 10.54	72.40 ± 10.58	0.13	77.40 ± 7.36	82.20 ± 8.18	73.13 ± 4.85	0.11
Spleen (g)	1.62 ± 0.53	1.66 ± 0.11	1.61 ± 0.23	0.96	2.63 ± 0.36	2.30 ± 0.23	2.55 ± 0.46	0.31
Lung (g)	37.95 ± 8.72	36.00 ± 5.60	39.07 ± 2.82	0.69	63.12 ± 4.63	52.05 ± 5.01	55.81 ± 4.17	0.0028
Kidneys (g)	12.86 ± 2.06	13.47 ± 2.38	13.75 ± 1.34	0.73	19.21 ± 2.01	19.22 ± 2.32	17.37 ± 1.24	0.13

*Note:* At 14 days after hypoxic‐iscemia (HI), no statistically significant differences were identified in body and organ weights among the groups; at 21 days after HI, significant differences were noted in body, brain, and lung weights among the groups (*p* < 0.0001, *p* = 0.0003, *p* = 0.0028, respectively). Furthermore, Tukey's multiple comparisons tests demonstrated that both the HI + NS and HI + hOPCs groups had lower body, brain, and lung weights than those in the control group. Additionally, the HI + hOPCs group exhibited a higher brain weight (*P* = 0.044) compared to the HI + NS group, although no significant differences were observed in body or lung weight. All data were analyzed with one–way ANOVA followed by Tukey's post hoc test. Control, twin siblings of HI fetuses; HI + NS, HI insult following normal saline administration; HI + hOPCs, HI insult following hOPCs administration.

Abbreviations: HI, hypoxic‐ischemia; NS, normal saline.

### 3.2 A Fetal Goat Model of PWMI Was Successfully Established

**FIGURE 2:**
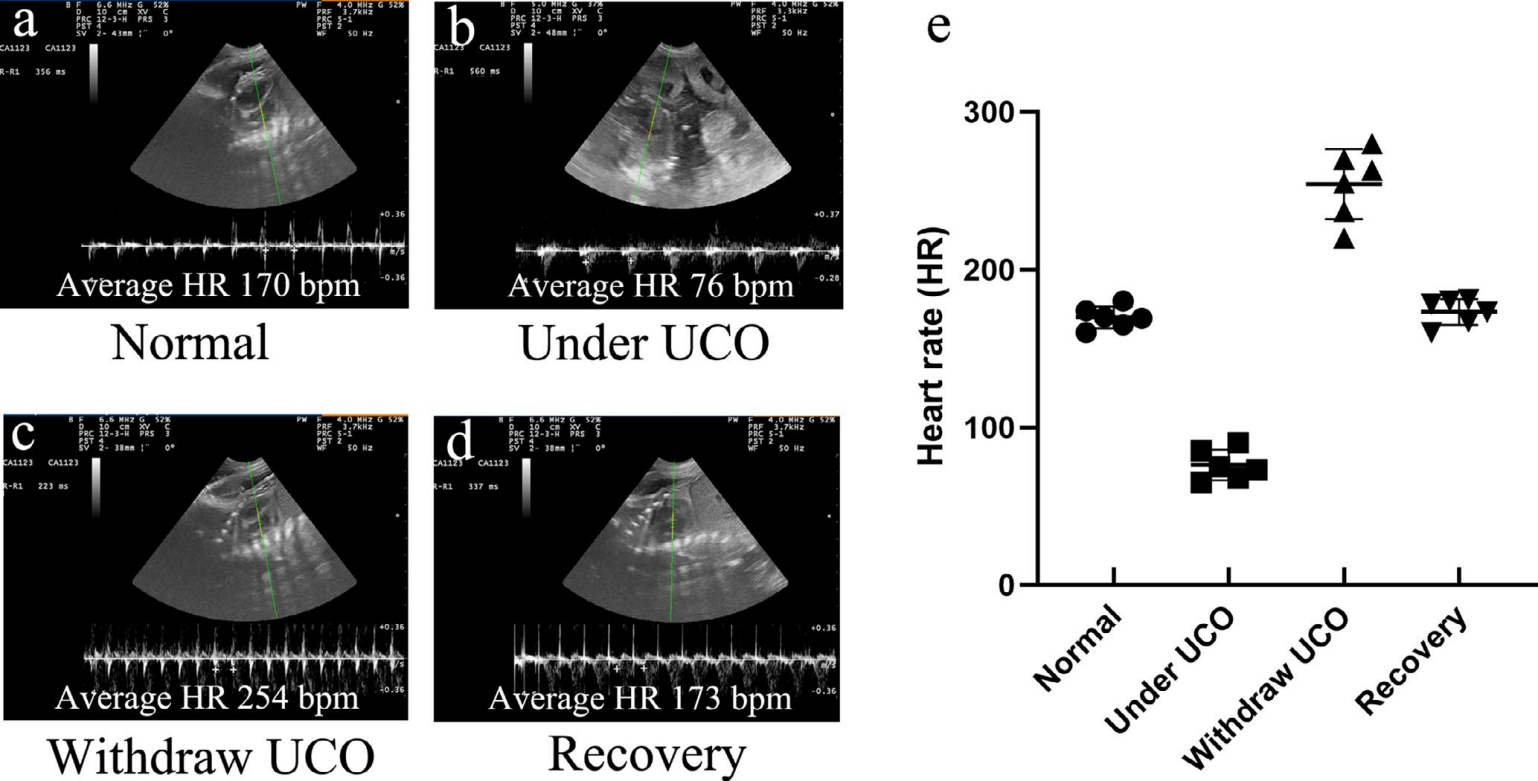
Fetal heart rate monitoring using ultrasound. (a) Normal heart rate of fetal goats is an average of approximately 170 beats per minute (bpm). (b) The fetal heart rate decreased to an average of 76 bpm within tens of seconds, and the amplitude of heart contractions was markedly reduced under umbilical cord occlusion (UCO). (c) The heart rate immediately increased to an average of 254 bpm for several seconds. (d) The heart rate returned around baseline after withdrawing UCO (within 1 min). (e) The line chart for the changes in fetal heart rate. *n* = 6. UCO, umbilical cord occlusion; HR, heart rate; bpm, beats per minute.

**FIGURE 3:**
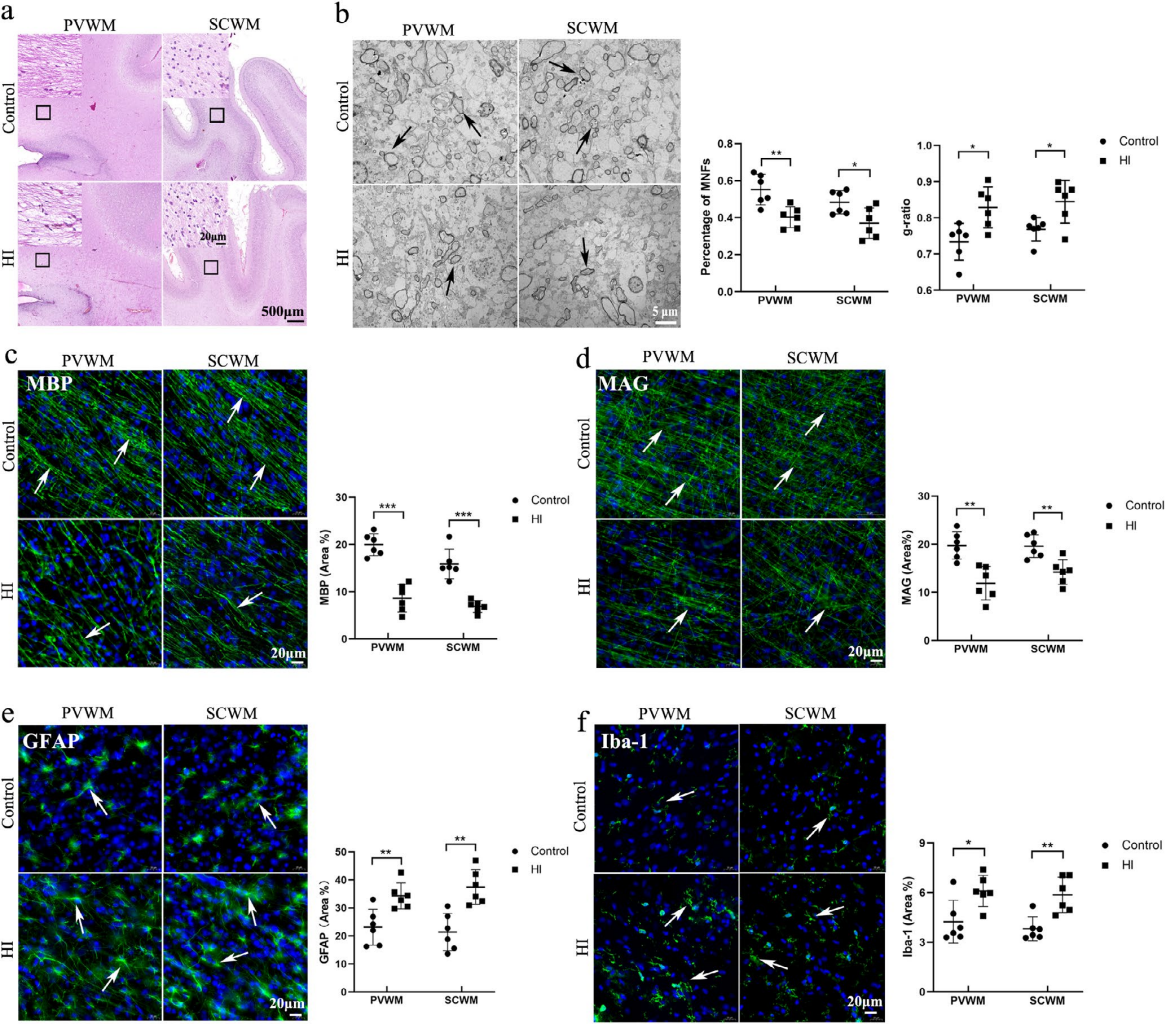
Hypoxic‐ischemia (HI) causes decreased myelination and increased neuroinflammation. Tissues were collected at Day 14 after HI. (a) Hematoxylin and eosin staining confirms that the HI group has disordered and loosely arranged white matter in PVWM and SCWM (Scale bar = 500 μm, 20 μm, *n* = 6 each group). (b) TEM demonstrates that the HI group has a reduced percentage of MNFs and a high g‐ratio. Arrows indicate myelin sheath (Scale bar = 5 μm, *n* = 6 each group, Mann–Whitney *U* test). (c) Immunofluorescent staining displays that the HI group has decreased MBP‐positive areas. Arrows represent MBP (Scale bar = 20 μm, *n* = 6 each group, Student's *t*‐test in PVWM, unpaired t‐test with Welch's correction in SCWM). (d) Immunofluorescent staining demonstrates that the HI group has decreased MAG‐positive areas. Arrows represent MAG (Scale bar = 20 μm, *n* = 6 each group, Student's *t*‐test). (e) Immunofluorescent staining reveals that the HI group had increased GFAP‐positive areas. Arrows indicate GFAP (Scale bar = 20 μm, *n* = 6 each group, Student's *t*‐test). (f) Immunofluorescent staining confirms that the HI group has increased Iba‐1‐positive areas. Arrows represent Iba‐1 (Scale bar = 20 μm, *n* = 6 each group, Student's *t*‐test in PVWM, Mann–Whitney *U* test in SCWM). *p < 0.05, **p < 0.01, ***p < 0.001. GFAP, glial fibrillary acidic protein; HI, hypoxic ischemia; Iba‐1, ionized calcium‐binding adaptor molecule‐1; MAG, myelin‐associated glycoprotein; MBP, myelin basic protein; MNFs, myelinated nerve fibers; PVWM, periventricular white matter; SCWM, subcortical white matter; TEM, transmission electron microscopy.

Assessments were conducted to verify whether the PWMI model was successfully induced in fetal goats. Ultrasound detection of fetal heart rate confirmed that the normal heart rate of fetal goats was approximately 170 bpm. When the umbilical cord was clamped, the fetal heart rate dropped to approximately 76 bpm within tens of seconds and the amplitude of heart contractions was markedly reduced, suggesting reduced peripheral perfusion. When the clamp was released, the instantaneous heart rate increased to approximately 254 bpm and returned to normal (within 1 min) (Figure 2). Additionally, H&E staining revealed no histopathological damage on Day 14 in the control group. However, the HI group had disordered and loosely arranged white matter in the PVWM and SCWM (Figure 3a). TEM revealed that the HI group had a lower percentage of MNFs than the control group (*p* = 0.004 for PVWM and *p* = 0.024 for SCWM) (Figure 3b). In addition, the g‐ratio (a small g‐ratio represents a thick myelin sheath layer) revealed that the HI group had a higher g‐ratio compared with the ratio observed in the control group (*p* = 0.026 for PVWM, *p* = 0.041 for SCWM), indicating a thin myelin sheath layer in the HI group. Immunofluorescence staining demonstrated that the HI group had decreased MBP‐positive (*p* < 0.001 for both PVWM and SCWM) and MAG‐positive areas (*p* = 0.002 for PVWM and *p* = 0.003 for SCWM) compared with the control group (Figure 3c,d), indicating a loss of myelin. Additionally, the HI group exhibited increased levels of activated astrocytes (GFAP^+^) (*p* = 0.006 for PVWM and *p* = 0.001 for SCWM) and microglia (Iba‐1^+^) (*p* = 0.016 for PVWM and *p* = 0.009 for SCWM) (Figure 3e,f), indicating an increase in inflammation. Collectively, these findings were consistent with the pathology of neonatal white matter injury, indicating that the PWMI model was successfully established.

### 3.3 hOPCs Were Able to Differentiate Into Mature Oligodendrocytes in the Brain

Fourteen days after transplantation, the presence of hOPCs in brains after HI + hOPCs was determined by observing CM‐DiI red fluorescent labeled hOPCs in the brain sections. CM‐DiI‐labeled hOPCs were detected in the brains of hOPC‐treated fetuses. Red fluorescence was not observed in the brains of animals treated with normal saline containing CM‐DiI red dye (Figure S2).

**FIGURE 4:**
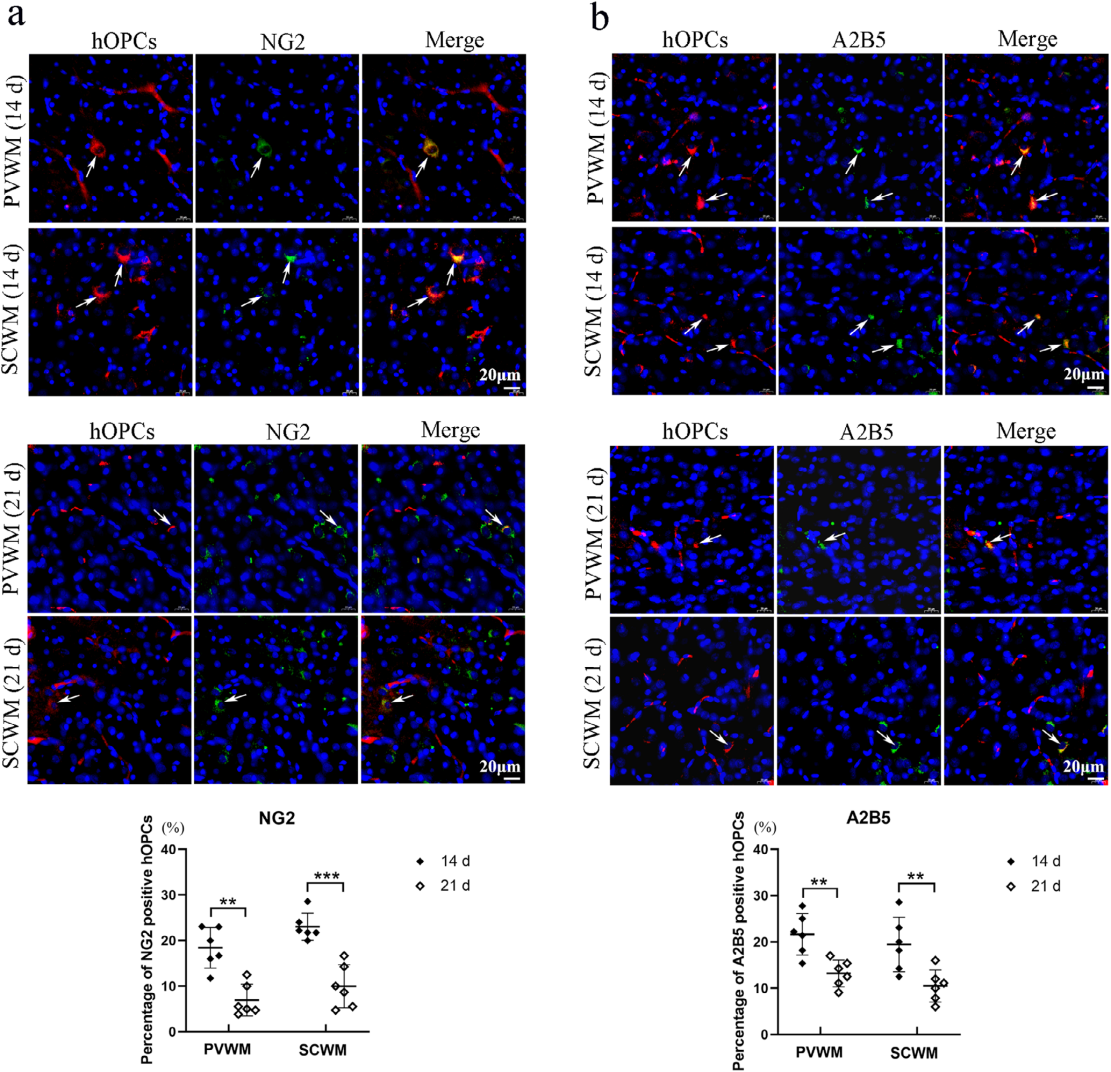
The percent of NG2 or A2B5 positive human oligodendrocyte precursor cells (hOPCs) were significant decreased at Day 21 compared with Day 14. (a) Immunofluorescence analysis showed that the percentages of NG2 positive hOPCs are 18.3% ± 4.2% (PVWM) and 23.0% ± 3% (SCWM) at Day 14, 6.9% ± 3.5% (PVWM) and 10.0% ± 4.7% (SCWM) at Day 21 (Scale bar = 20 μm, *n* = 6 at each time point. Mann–Whitney *U* test in PVWM, Student's *t*‐test in SCWM). (b) Immunofluorescence analysis showed that the percentage of A2B5 positive hOPCs are 21.7% ± 4.5% (PVWM) and 19.4% ± 6% (SCWM) at Day 14, 13.2% ± 3.0% (PVWM) and 10.5% ± 3.7% (SCWM) at day 21 (Scale bar = 20 μm, *n* = 6 at each time point, Student's *t*‐test). **p < 0.01, ***p < 0.001. PVWM, periventricular white matter; SCWM, subcortical white matter.

The expression of NG2, a marker used to identify preoligodendrocytes, was detected using immunofluorescence staining. The percentages of NG2 positive hOPCs were 18.3% ± 4.2% (PVWM) and 23.0% ± 3% (SCWM) at Day 14, 6.9% ± 3.5% (PVWM) and 10.0% ± 4.7% (SCWM) at day 21 respectively (Figure 4a). Significant differences were identified in the percentages of NG2‐positive hOPCs in the PVWM (*p* = 0.004) and SCWM (*p* < 0.001) between Days 14 and 21. Immunofluorescence staining for A2B5, a preoligodendrocyte marker, was performed. The percentage of A2B5 positive hOPCs were 21.7% ± 4.5% (PVWM) and 19.4% ± 6% (SCWM) at day 14, 13.2% ± 3.0% (PVWM) and 10.5% ± 3.7% (SCWM) at Day 21 (Figure 4b). Significant differences were in the percentage of A2B5 positive hOPCs in the PVWM (*p* = 0.003) and SCWM (*p* = 0.009) between Days 14 and 21.

**FIGURE 5:**
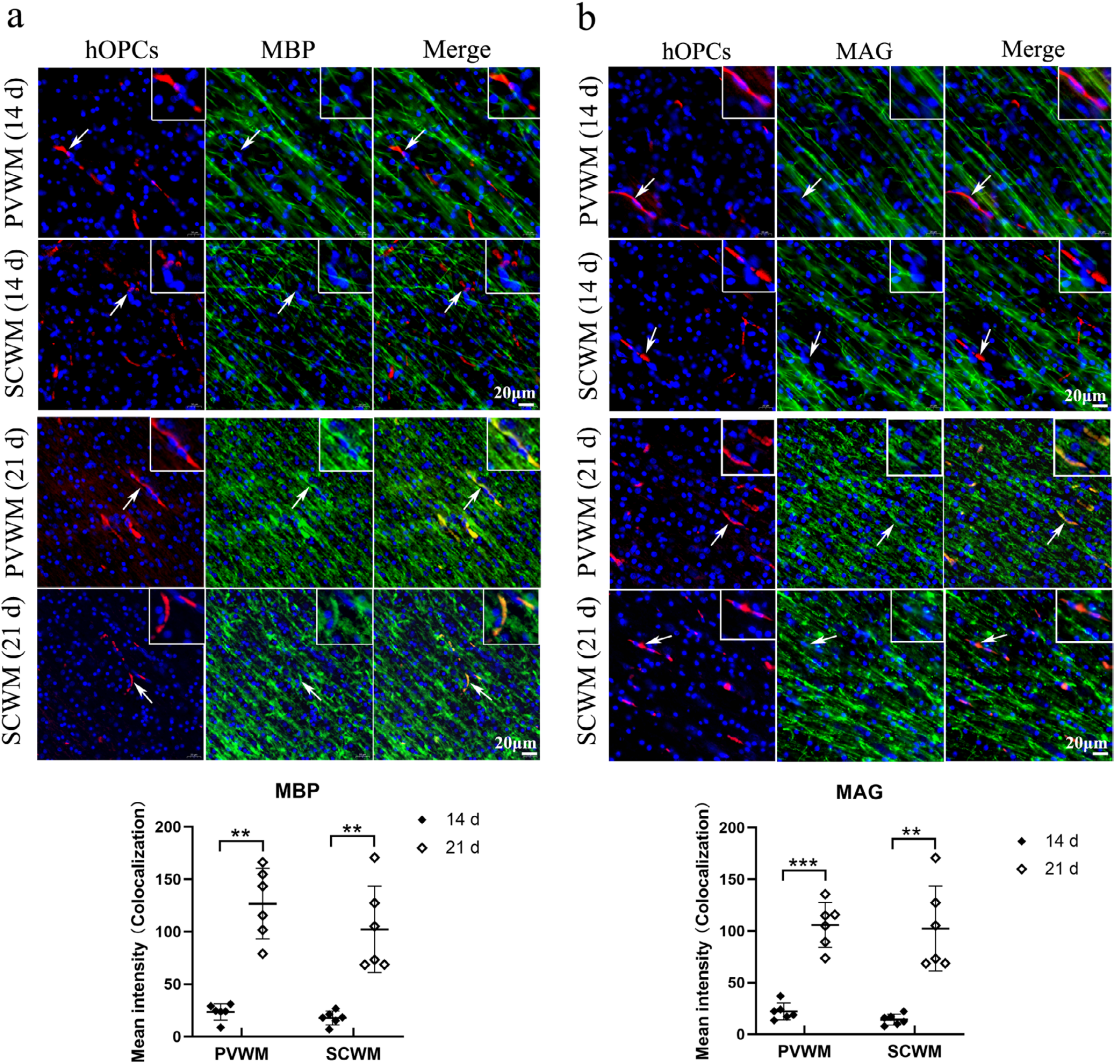
Human oligodendrocyte precursor cells (hOPCs) differentiated into mature oligodendrocytes at Day 21. Immunofluorescent staining showed that transplanted hOPCs expressed markers of mature oligodendrocytes (MBP and MAG) at Day 21. (a) CM‐DiI labeled hOPCs (red) barely expressed MBP (green) at Day 14 but strongly expressed MBP at Day 21 both in SCWM and PVWM (Scale bar = 20 μm, *n* = 6 at each time point. Mann–Whitney *U* test in PVWM; unpaired *t*‐test with Welch's correction in SCWM) (b) Fluorescence co‐localization demonstrates that CM‐DiI labeled hOPCs barely expressed MAG (green) at Day 14 but strongly expressed MAG at Day 21 both in SCWM and PVWM. The arrows indicate hOPCs (Scale bar = 0 μm. *n* = 6 at each time point, unpaired t‐test with Welch's correction). ***p < 0.001. MAG, myelin‐associated glycoprotein; MBP, myelin basic protein; PVWM, periventricular white matter; SCWM, subcortical white matter.

The expression of MBP and MAG (two representative markers of mature oligodendrocytes) were detected at Days 14 and 21 after administration to observe the differentiation ability of hOPCs. Fluorescence co‐localization revealed that CM‐DiI labeled hOPCs (red fluorescence) barely expressed MBP or MAG (green fluorescence) at Day 14 but strongly expressed MBP and MAG at Day 21 in both the PVWM and SCWM regions (Figure 5). These results suggested that hOPCs showed a differentiation ability in the brain.

### 3.4 Intranasal Administration of hOPCs Alleviating WMI

**FIGURE 6:**
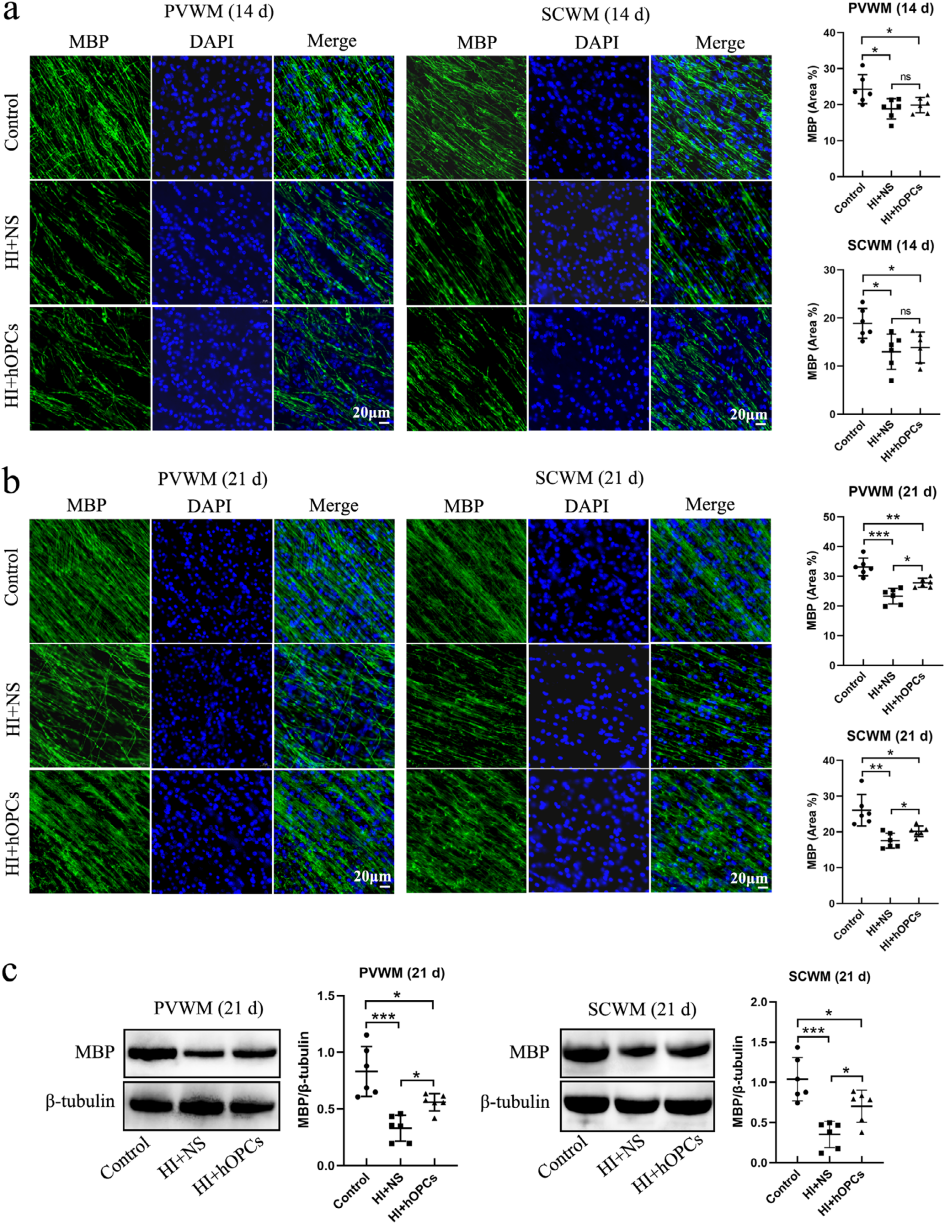
Human oligodendrocyte precursor cells (hOPCs) administration increased the level of myelin basic protein (MBP) at Day 21 after hypoxic‐ischemia (HI). Immunofluorescence staining and immunoblotting showed that HI resulted in decreased MBP expression compared with control group. However, hOPCs administration increased the level of MBP at Day 21, but not at Day 14. (a) At Day 14, immunofluorescence staining reveals that HI + NS group has decreased MBP‐positive areas both in SCWM and PVWM compared with control. However, no significant difference is identified between the HI + NS and HI + hOPCs groups (Scale bar = 0 μm, *n* = 6 for each group, one–way ANOVA followed by Tukey's post hoc test). (b) At Day 21, the MBP‐positive areas in HI + NS group are lower than that in control group both in the SCWM and PVWM regions. The MBP‐positive areas in HI + hOPCs are higher than that in the HI + NS group, but still lower than that in the control group (Scale bar = 0 μm, *n* = 6 for each group, one‐way ANOVA followed by Tukey's post hoc test in PVWM; Welch ANOVA test in SCWM). (c) Immunoblotting confirms that at Day 21, HI resulted in decreased levels of MBP in the SCWM and PVWM regions. The level of MBP in HI + hOPCs is higher than that in the HI + NS group, but still lower than that in the control group in the SCWM and PVWM regions (*n* = 6 for each group, one–way ANOVA followed by Tukey's post hoc test). ns = not significant, *p < 0.05, **p < 0.01, ***p < 0.001. Control, twin siblings of HI fetuses; HI + NS, HI insult following normal saline administration; HI + hOPCs, HI insult following hOPCs administration. HI, hypoxic‐ischemia; MBP, myelin basic protein; NS, normal saline; PVWM, periventricular white matter; SCWM, subcortical white matter.

The expression of MBP in the SCWM or PVWM was examined using immunofluorescence staining. On Day 14, the percentage of MBP‐positive areas in the PVWM and SCWM were significantly reduced after HI compared with that in the control group (*p* = 0.021 and *p* = 0.020, respectively). However, no significant differences were observed between the HI + NS and HI + hOPC groups (*p* = 0.83 in PVWM, *p* = 0.89 in SCWM) (Figure 6a). On Day 21, HI resulted in reduced MBP‐positive areas in the PVWM and SCWM (*p* < 0.001 and *p* = 0.008, respectively). Additionally, HI + hOPCs increased the percentage of MBP‐positive areas compared with those in the HI + NS group in the PVWM and SCWM (*p* = 0.017 and *p* = 0.041, respectively). However, fewer MBP‐positive areas were observed in HI + hOPCs than in the control group (*p* = 0.005 and *p* = 0.047, respectively) (Figure 6b). The levels of MBP in the PVWM and SCWM at Day 21 were measured by immunoblotting, which confirmed that the HI + NS group had significantly lower levels of MBP than the control group (*p* < 0.001 for both PVWM and SCWM). Furthermore, the HI + hOPCs group had increased MBP levels compared with the HI + NS group in both the PVWM (*p* = 0.046) and SCWM (*p* = 0.033) regions; however, the levels were lower than that in the control group (*p* = 0.017 and *p* = 0.041, respectively) (Figure 6c).

**FIGURE 7:**
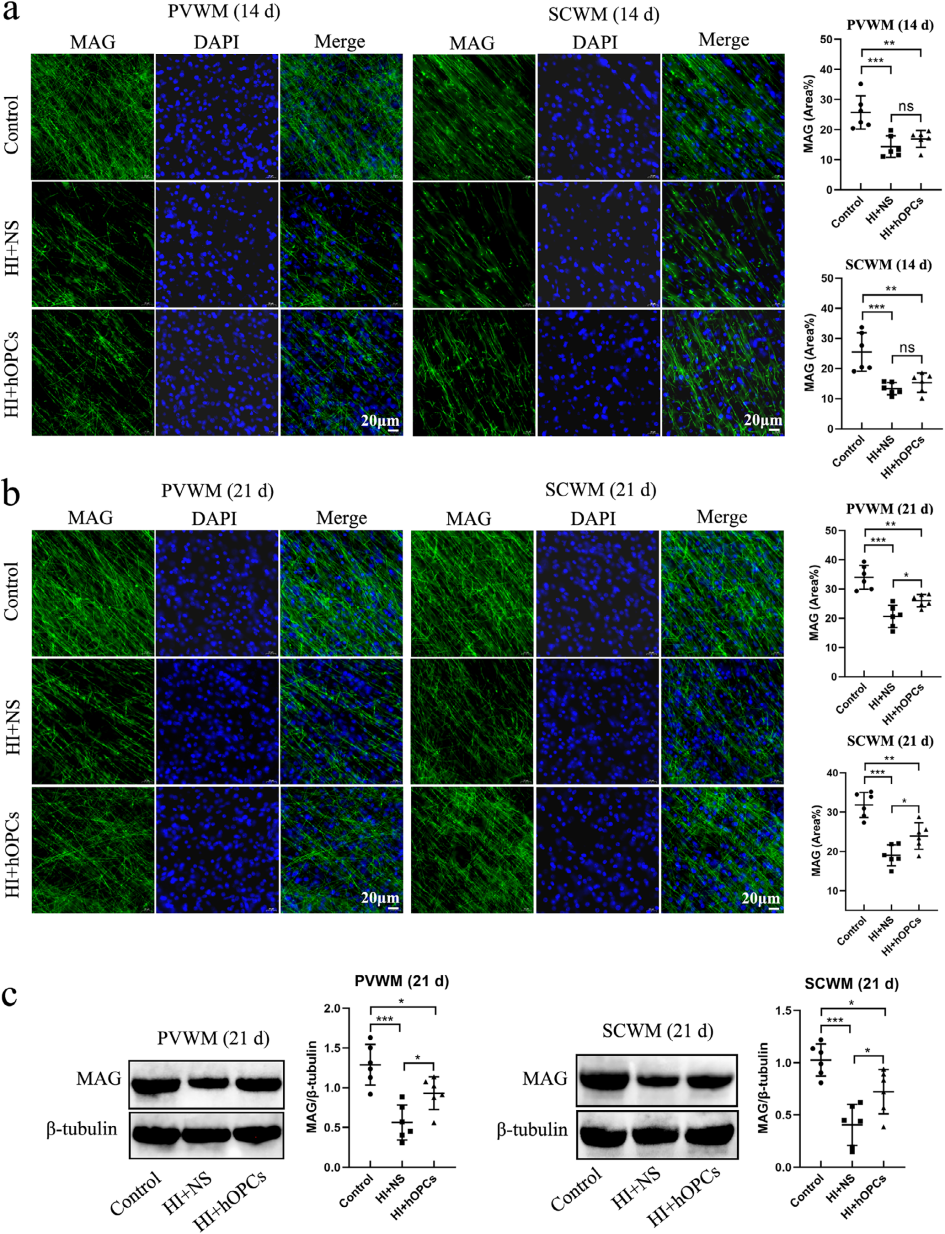
Human oligodendrocyte precursor cells (hOPCs) administration increased the level of myelin‐associated glycoprotein (MAG) at Day 21 after hypoxic‐ischemia (HI). Immunofluorescence staining and immunoblotting showed that HI resulted in decreased MAG expression compared with control group. However, hOPCs administration increased the level of MAG at Day 21, but not at Day 14. (a) At Day 14, immunofluorescence staining confirms that HI resulted in decreased MAG‐positive areas. No significant differences were identified between the HI + NS and HI + hOPCs groups both in the SCWM and PVWM regions (Scale bar = 20 μm, *n* = 6 for each group). (b) At Day 21, HI results in a decreased MAG‐positive area. The MAG‐positive area in HI + hOPCs is higher than that in the HI + NS group but lower than that in the control group in the SCWM and PVWM regions (Scale bar =20 μm, *n* = 6 for each group). (c) Immunoblotting reveals that at Day 21, HI results in a decreased level of MAG. The level of MAG in HI + hOPCs is higher than in the HI + NS group but still lower than that in the control group both in the SCWM and PVWM regions. (*n* = 6 for each group). ns = not significant, *p < 0.05, **p < 0.01, ***p < 0.001. All data were analyzed using one‐way ANOVA followed by Tukey's post hoc test. Control, twin siblings of HI fetuses; HI + NS, HI insult following normal saline administration; HI + hOPCs, HI insult following hOPCs administration. HI, hypoxic ischemia; MAG, myelin‐associated glycoprotein; NS, normal saline; PVWM, periventricular white matter; SCWM, subcortical white matter.

The expression of MAG in each group was detected using immunofluorescence staining. At Day 14, the percentage of MAG‐positive areas was significantly reduced after HI in both PVWM (*p* < 0.001) and SCWM (*p* < 0.001) regions. However, no significant difference was identified between the HI + NS and HI + hOPC groups (*p* = 0.55 in PVWM, *p* = 0.71 in SCWM) (Figure 7a). On Day 21, the HI insult resulted in reduced MAG‐positive areas compared with that in the control group (*p* < 0.001 for both PVWM and SCWM). Additionally, hOPCs administration after HI increased the percentage of MAG‐positive areas compared with the areas in the HI + NS group in both the PVWM and SCWM regions (*p* = 0.039 and *p* = 0.037, respectively). However, fewer MAG‐positive areas were observed in HI + hOPCs group compared with the control group (*p* = 0.003 and *p* = 0.001, respectively) (Figure 7b). The levels of MAG in the PVWM and SCWM at Day 21 were analyzed by immunoblotting. Compared with the control group, the HI + NS group had significantly lower MAG levels (*p* < 0.001). Following HI, hOPCs administration resulted in an increased MAG level compared to that in the HI + NS group in both PVWM (*p* = 0.035) and SCWM (*p* = 0.027) regions but the levels were lower than that in the control group (*p* = 0.040 and *p* = 0.036, respectively) (Figure 7c).

**FIGURE 8:**
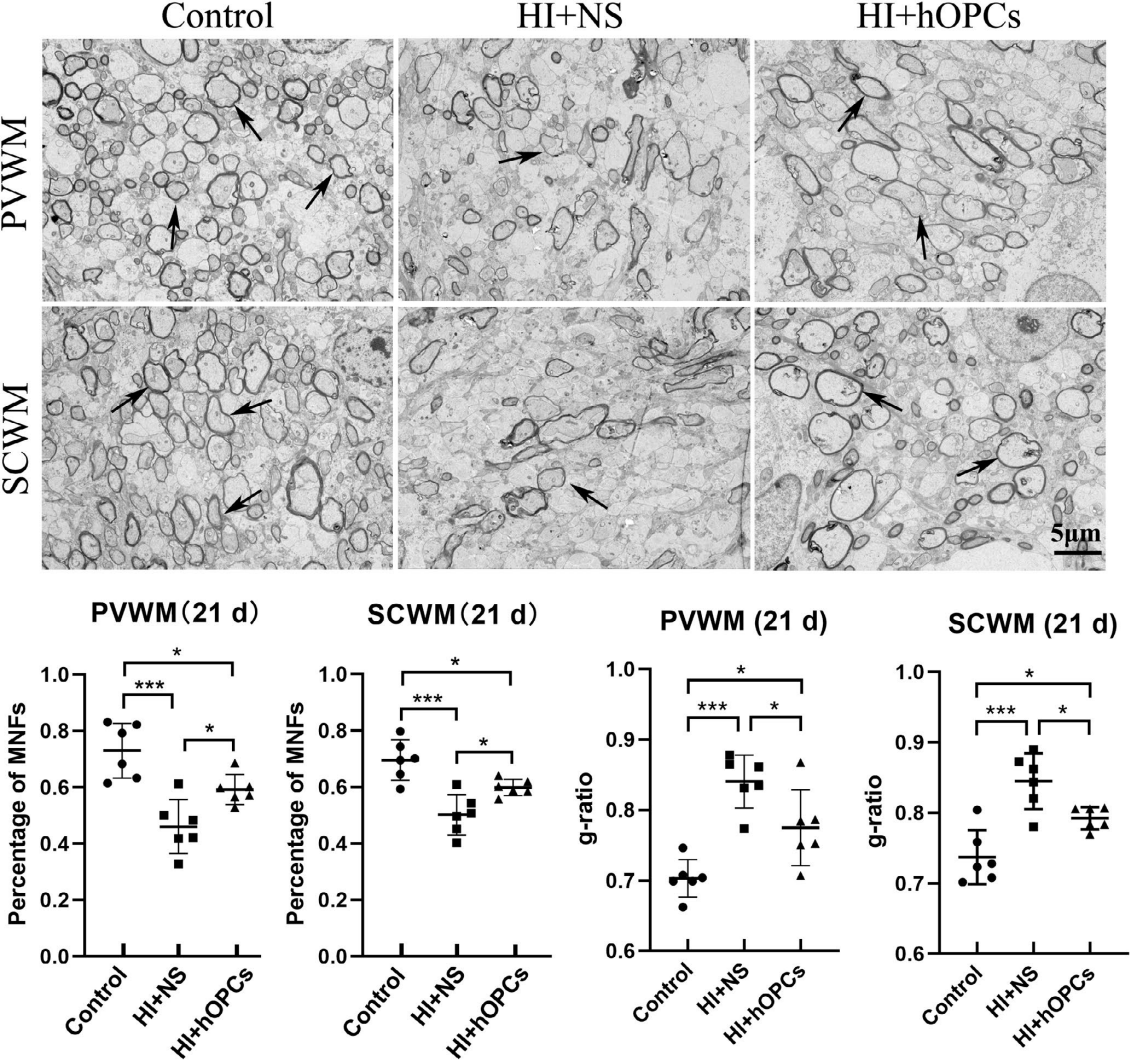
Human oligodendrocyte precursor cells (hOPCs) administration increased the percentage of myelinated nerve fibers (MNFs) and thickness of myelin sheath at Day 21 after the hypoxic‐ischemia (HI) insult. In both PVWM and SCWM, HI decreases the percentage of MNFs. The HI + hOPCs group has a higher percentage of MNFs compared with that in the HI + NS group, which is lower than that in the control group in the PVWM and SCWM regions. Arrows indicate MNFs. In both PVWM and SCWM, HI + NS results in an increased g‐ratio compared with the ratio observed in the control group. The HI + hOPC group had a decreased g‐ratio compared with the HI + NS group. The HI + hOPC group still has a higher g‐ratio than the control group in the PVWM and SCWM regions. Arrows represent the myelin sheath. Scale bar = 5 μm. *n* = 6 for each group. The brain tissue was collected at Day 21 after HI. *p < 0.05, **p < 0.01, ***p < 0.001. All data were analyzed with one–way ANOVA followed by Tukey's post hoc test. Control, twin siblings of HI fetuses; HI + NS, HI insult following normal saline administration; HI + hOPCs, HI insult following hOPCs administration. HI, hypoxic ischemia; MNFs, myelinated nerve fibers; NS, normal saline; PVWM, periventricular white matter; SCWM, subcortical white matter.

The ultrastructure of the myelin sheath in PVWM and SCWM (21 days) were observed using TEM. The percentage of MNFs decreased after HI in both PVWM and SCWM (*p* < 0.001 for both PVWM and SCWM). After HI, hOPC administration resulted in an increased percentage of MNFs compared to that in the HI + NS group (*p* = 0.042 for PVWM, *p* = 0.037 for SCWM) but the percentage of MNFs were lower than that in the control group (*p* = 0.033 for PVWM, *p* = 0.036 for SCWM) (Figure 8). Furthermore, HI + NS resulted in an increased g‐ratio compared with that in the control group (*p* < 0.001 for both PVWM and SCWM) indicating a thin myelin sheath. The HI + hOPC group had a lower g‐ratio than the ratio in the HI + NS group (*p* = 0.035 for PVWM and *p* = 0.038 for SCWM), but the HI + hOPC group had a higher g‐ratio than that in the control group (*p* = 0.021 for PVWM and *p* = 0.029 for SCWM) indicating that the HI + hOPC group had a thicker myelin sheath than the HI + NS group, but had a thinner myelin sheath than the control group. Therefore, intranasal administration of hOPCs can increase the percentage of MNFs and thickness of the myelin sheath after HI insult.

### 3.5 Intranasal Administration of hOPCs Did Not Aggravate Inflammatory Response After HI Insult

**FIGURE 9:**
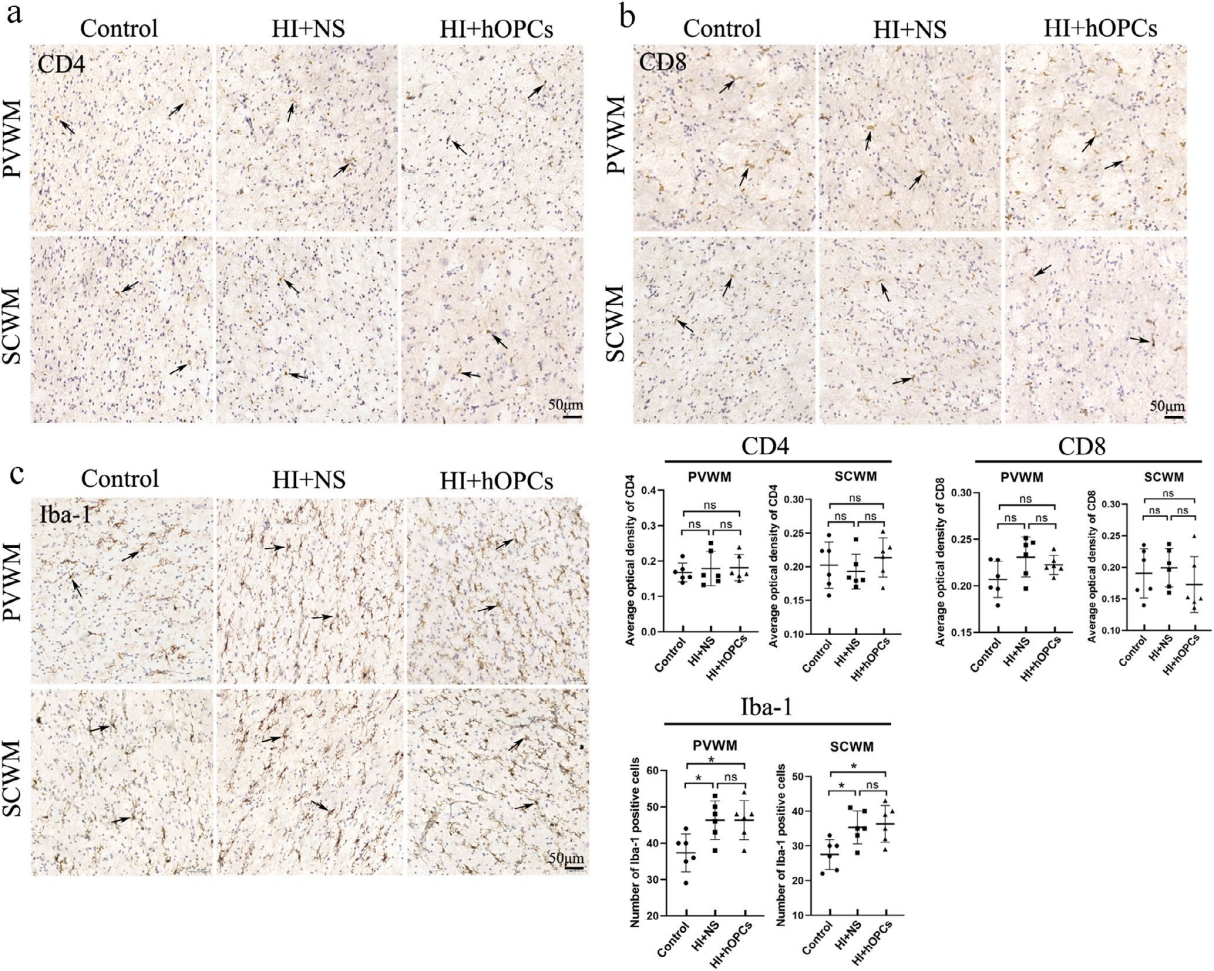
Intranasal administration of human oligodendrocyte precursor cells (hOPCs) did not increase inflammatory cells after hypoxic‐ischemia (HI) insult. (a) Immunohistochemistry analysis for CD4 shows no significant differences in the control, HI + NS, and HI + hOPCs groups. Arrows indicate CD4. (b) Immunohistochemistry analysis for CD8 show no significant differences in the control, HI + NS, and HI + hOPCs groups. Arrows indicate CD8. (c) Immunohistochemistry analysis for Iba‐1 confirms that HI results in an increased number of Iba‐1 positive cells. No differences were observed between the HI + NS and HI + hOPCs groups. Arrows indicate Iba‐1. Scale bar = 50 μm. *n* = 6 for each group. Brain tissue was collected 21 days after HI. ns = not significant, *p < 0.05. All data were analyzed with one–way ANOVA followed by Tukey's post hoc test. Control, twin siblings of HI fetuses; HI + NS, HI insult following normal saline administration; HI + hOPCs, HI insult following hOPCs administration. HI, hypoxic ischemia; Iba‐1, ionized calcium‐binding adaptor molecule‐1; NS, normal saline; PVWM, periventricular white matter; SCWM, subcortical white matter.

**FIGURE 10:**
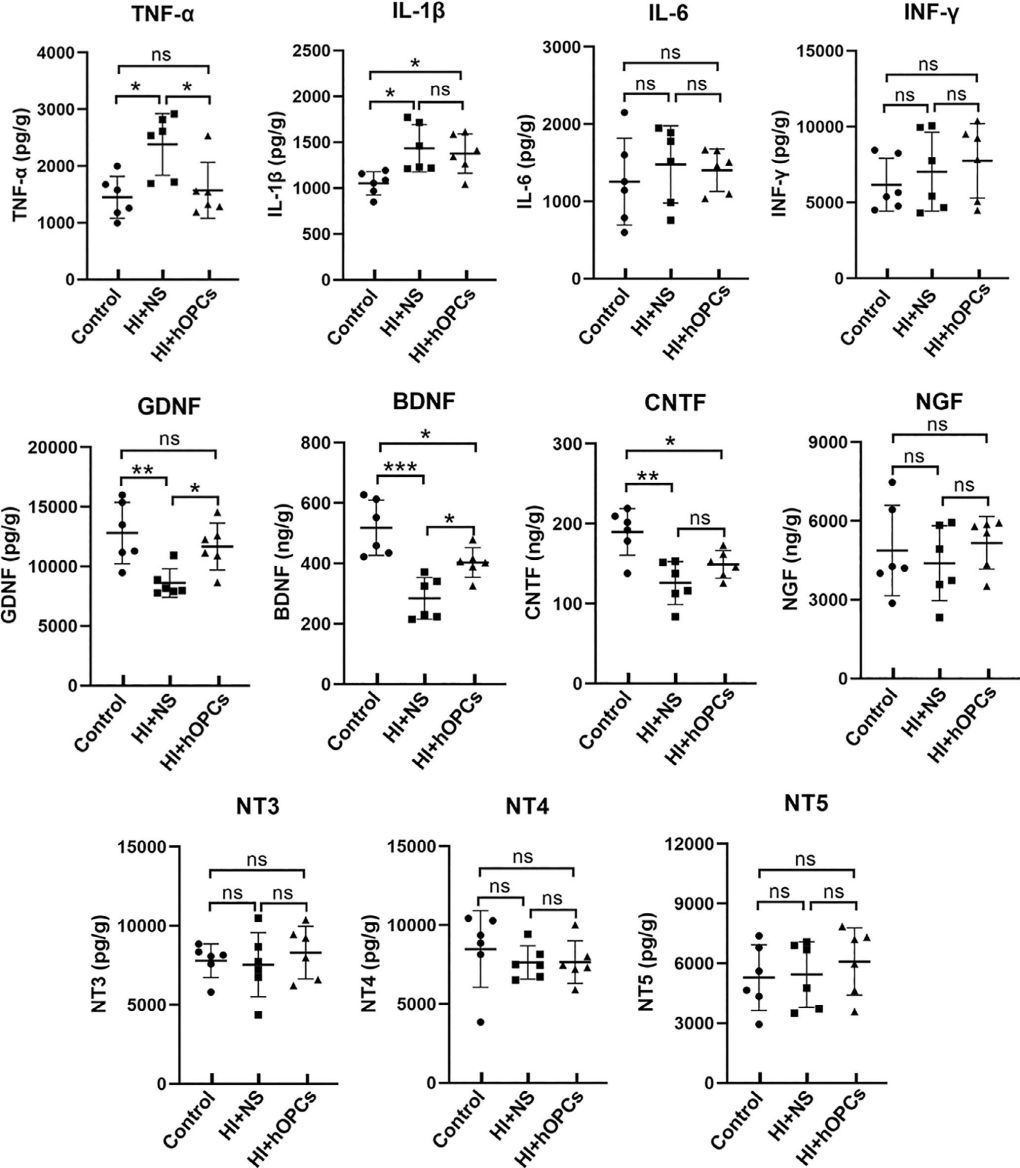
Human oligodendrocyte precursor cells (hOPCs) administration reduced the level of tumor necrosis factor‐alpha (TNF‐α) and increased the levels of glial‐derived neurotrophic factor (GDNF), brain‐derived neurotrophic factor (BDNF) after hypoxic‐ischemia. The concentrations of inflammatory factors (TNF‐α, IL‐1β, IL‐6, and INF‐γ) and neurotrophic factors (GDNF, BDNF, CNTF, NGF, NT‐3, NT‐4, and NT‐5) in white matter are determined using enzyme‐linked immunosorbent assay (ELISA). Regarding the inflammatory factors, HI significantly increases the levels of TNF‐α and IL‐1β compared with that in the control group. HI + hOPCs has lower levels of TNF‐α but not IL‐1β compared with that in the HI + NS group. No differences were identified in the groups in the levels of IL‐6 and INF‐γ. Regarding neurotrophic factors, the HI + NS group has decreased levels of GDNF, BDNF, and CNTF compared with that in the control group. HI + hOPCs had increased levels of GDNF and BDNF but not CNTF compared with that in the HI + NS group. No significant differences were observed in the NGF, NT‐3, NT‐4, or NT‐5 levels among the groups. *n* = 6 for each group. Brain tissue was collected at Day 21 after HI. ns = not significant, *p < 0.05, **p < 0.01, ***p < 0.001. All data were analyzed with one–way ANOVA followed by Tukey's post hoc test. Control, twin siblings of HI fetuses; HI + NS, HI insult following normal saline administration; HI + hOPCs, HI insult following hOPCs administration. BDNF, brain‐derived neurotrophic factor; CNTF, ciliary neurotrophic factor; GDNF, glial‐derived neurotrophic factor; HI, hypoxic‐ischemia; IL‐1β, interleukin‐1 beta; IL‐6, interleukin 6; INF‐γ, interferon‐gamma; NGF, nerve growth factor; NS, normal saline; NT‐3, neurotrophin‐3; NT‐4, neurotrophin‐4; NT‐5, neurotrophin‐5; TNF‐a, tumor necrosis factor‐alpha.

At Day 21, inflammatory cells (CD4, CD8, and Iba‐1) were detected using immunohistochemistry and the concentration of inflammatory factors (TNF‐α, IL‐1β, IL‐6, INF‐γ) were determined using ELISA. The CD4 and CD8 were not significantly different in the control group, HI + NS, and HI + hOPCs groups (Figure 9a,b). Iba‐1 positive cells were higher in the HI + NS group compared with that in the control group (*p* = 0.026 for PVWM, *p* = 0.032 for SCWM). HI + hOPCs had an increased number of Iba‐1 positive cells compared with cells in the control group (*p* = 0.026 for PVWM, *p* = 0.016 for SCWM). No differences were identified between the HI + NS and HI + hOPCs groups (Figure 9c), indicating that the intranasal administration of hOPCs did not further increase the number of inflammatory cells after HI. Furthermore, inflammatory factors detected by ELISA (Figure 10) revealed that HI significantly increased the levels of TNF‐α (*p* = 0.010) and IL‐1β (*p* = 0.015) compared with that in the control group. HI + hOPCs had lower levels of TNF‐α compared with the levels in the HI + NS group (*p* = 0.025). No differences were observed between the HI + hOPCs and control groups (*p* = 0.89). HI + hOPCs had higher levels of IL‐1β compared with that in the control group (*p* = 0.040), but no significant differences were detected when compared with HI + NS group (*p* = 0.88). No differences were observed between the groups in the levels of IL‐6 and INF‐γ. These results indicated that HI caused an increased inflammatory response; however, intranasal administration of hOPCs did not aggravate the inflammatory response after HI. In addition, hOPCs administration reduced TNF‐α levels.

### 3.6 Intranasal Administration of hOPCs Increased the Levels of GDNF and BDNF

The concentrations of classic neurotrophic factors (GDNF, BDNF, CNTF, NGF, NT‐3, NT‐4, and NT‐5) in the white matter at Day 21 after HI were detected using ELISA (Figure 10). The results confirmed that the HI + NS group had decreased levels of GDNF (*p* = 0.007), BDNF (*p* < 0.001), and CNTF (*p* = 0.001) compared with the control group. However, HI + hOPCs showed increased levels of GDNF (*p* = 0.044) and BDNF (*p* = 0.030) but not CNTF (*p* = 0.267) compared with that in the HI + NS group. The HI + OPC group had lower BDNF levels than that in the control group (*p* = 0.036). No significant differences in the level of GDNF was observed between the HI + hOPCs and control groups (*p* = 0.607). Additionally, no significant differences were observed in NGF, NT‐3, NT‐4, or NT‐5 levels among the groups.

### 3.7 Safety Evaluation

At Day 21, immunofluorescence staining demonstrated that the administered hOPCs barely expressed Ki67 (mean ± SD 3.9% ± 3.2%) indicating a low capacity of proliferation in vivo (Figure S3). On Day 21, CM‐DiI‐labeled hOPCs were not observed in the liver, spleen, heart, lungs, or kidneys (Figure S4). Blood test results confirmed no significant differences between the groups (Table 2).

**TABLE 2:** Blood tests among groups at 21 days after hypoxic‐ischemia.

	Control (*n* = 6, Mean ± SD)	HI + NS (*n* = 6, Mean ± SD)	HI + hOPCs (*n* = 6, Mean ± SD)	*p*
ALT (U/L)	14.00 ± 6.54	18.00 ± 4.60	11.83 ± 5.19	0.18
AST (U/L)	13.33 ± 6.38	14.33 ± 5.50	9.33 ± 2.94	0.22
CREA (μmol/L)	101.97 ± 28.95	93.00 ± 30.03	102.90 ± 30.32	0.82
RBC (10^12^/L)	7.00 ± 1.88	7.31 ± 1.29	7.05 ± 1.56	0.94
HCT (%)	37.37 ± 3.04	37.28 ± 1.72	39.53 ± 3.10	0.29
MCHC (g/dL)	24.90 ± 3.96	24.52 ± 3.26	24.28 ± 4.99	0.97
WBC (10^9^/L)	4.92 ± 1.94	7.18 ± 1.69	5.42 ± 2.30	0.15
PLT (10^9^/L)	469.00 ± 49.64	448.83 ± 77.38	519.50 ± 89.20	0.85

*Note:* No significant difference was observed in blood test results among groups. All data were analyzed with one–way ANOVA followed by Tukey's post hoc test. Control, twin siblings of HI fetuses; HI + NS, HI insult following normal saline administration; HI + hOPCs, HI insult following hOPCs administration.

Abbreviations: ALT, alanine transaminase; AST, aspartate transaminase; CREA, creatinine; HCT, hematocrit; HI, hypoxic ischemia; MCHC, mean corpuscular hemoglobin concentration; NS, normal saline; PLT, platelets; RBC, red blood cell; WBC, white blood cell.

## 4 Discussion

Although in vitro experiments and small animal models offer valuable insights, large animal models can accurately predict drug administration or treatment in humans because of their close anatomical and physiological similarities with humans. Research involving large animals helps bridge the knowledge gap between laboratory studies and human applications, thereby enhancing the translation efficiency of research results. This study is the first to establish a PWMI model in fetal goats. Transnasal hOPCs can differentiate into mature oligodendrocytes in the brain tissue and improve the proportion of myelinated nerve fibers and thickness of the myelin sheath in the goat PWMI model. This aids in determining information such as the dose, time, and route of hOPCs administration. Moreover, this study provides preliminary clinical evidence for the safety and efficacy of hOPC transplantation for the treatment of PWMI.

PWMI models form the basis for research for assessing the pathogenesis and treatment strategies for WMI in preterm infants. The application of large animal models to pathophysiological and clinical translational research is particularly attractive. In this study, a fetal goat model of PWMI was established using UCO at a gestational age of 100–105 days (term: 145 days) (similar to the 24–28 weeks of gestational age in humans). At this gestational age, OPCs are highly vulnerable to HI [12]. The fetal goats and sheep share similar neurodevelopmental processes. Therefore, we used a goat model of PWMI and conducted the experiments referring mostly to the protocol for a sheep model of PWMI. Some steps were modified during the procedure. A fetal sheep model of PWMI is established by inducing 25 min of HI through complete UCO [14, 15, 16, 17]. However, in fetal goats, we discovered that 25 min of continuous UCO resulted in the disappearance of the fetal heart rate within a few hours after HI. After repeated attempts, we observed that intermittent UCO (5 min × 5) greatly reduced fetal death. The results of our study demonstrated disordered and loosely arranged white matter and significantly decreased mature myelin sheaths in the brains of fetal goats subjected to intermittent UCO confirming the successful establishment of a fetal goat model of PWMI. Moreover, an important advantage of fetal goats in this model is that pregnant goats usually carry two fetuses. Therefore, we could use one fetus as the experimental group and the other as the control group, so that the results were comparable.

Administration of OPCs is one of the most promising therapeutic strategies for treating PWMI. OPCs exhibit favorable migratory and differentiation properties. Research has indicated that exogenous OPCs can integrate into the brain tissue of the receptor and further mature into myelinated oligodendrocytes, thereby wrapping around axons to restore the structure of the white matter [29]. Previous studies have shown that exogenous OPCs can produce matrix metalloproteinase 9, an enzyme that increases the permeability of the blood–brain barrier, enhancing the ability of OPCs to penetrate brain tissues [30]. Lateral ventricular injection of hOPCs has been demonstrated to restore the white matter structure, increase sheath thickness, and improve learning and memory ability in a rat model of WMI [8, 11]. In our study, we discovered that exogenous hOPCs could survive in the brain. Moreover, using antibodies against preoligodendrocytes (NG2 and A2B5) and mature oligodendrocytes (MBP and MAG), hOPCs differentiated into mature oligodendrocytes and alleviated white matter injury in a fetal goat model of PWMI.

Regarding the administration route of donor cells, systemic administration (such as arteriovenous administration) and local administration (such as intraventricular puncture administration and intranasal administration) have both been studied in WMI models [19, 24, 31, 32]. However, arteriovenous administration of cells carries a risk of microthrombus formation and donor cells have difficulty crossing the blood–brain barrier [19]. Intraventricular puncture is an invasive procedure that can potentially damage brain tissues. Intranasal delivery of cells into the brain has been underexplored for decades [21, 33]. Numerous studies have demonstrated that cells delivered intranasally target the central nervous system and are distributed throughout the brain in animal models [21, 22, 24, 32, 34]. Additionally, intranasal administration of mesenchymal stem cells in a rat WMI model improved myelin reduction [24]. In a mouse model of HI brain injury, intranasal administration of mesenchymal stem cells alleviated the brain injury and improved cognitive function [34]. Moreover, intranasal administration of human amniotic epithelial cells reduced the number of inflammatory cells in the brain and improved brain injury in a fetal sheep model of WMI [14]. The intranasal pathway is a noninvasive method of administration making the pathway well‐suited for unstable and fragile infants when compared with other routes. A clinical trial (NCT03356821) was conducted in the Netherlands to evaluate the safety of intranasal mesenchymal stem cell administration in neonates with perinatal arterial stroke. Therefore, considering the feasibility and safety of the clinical application of cell administration in PWMI, intranasal administration of hOPCs was used in the present study.

The number of cells used for the treatment differed according to the route of administration. In a fetal sheep PWMI model, the intravenous delivery of 1–5 × 10^8^ stem cells protected preterm white matter brain development from HI [19, 35]. However, intranasal administration requires fewer donor cells than systemic administration. For instance, the intranasal infusion of 4 × 10^7^ human amniotic epithelial cells improved white matter maturation after HI [14]. A clinical trial (NCT03356821) used 5 × 10^7^ mesenchymal stem cells administered via the intranasal route to treat neonatal perinatal arterial stroke. Therefore, 2 × 10^7^ hOPCs were administered intranasally in this study.

Compared to adults, the immune system of newborns is immature, facilitating the induction of immune tolerance [36]. In newborn mice, naive T cells usually produce a Th2 immune response. Compared with the pro‐inflammatory Th1 and Th17 immune responses, the Th2 immune response helps to protect donor cells against immune rejection [37]. Additionally, the thymus plays an important role in immune tolerance. Studies have shown that the removal of the thymus causes acute cell rejection and leads to failure in tolerance induction. The presence of thymus in newborns enables immune tolerance [37]. In this study, intranasal administration of hOPCs did not increase inflammatory response following HI insult, indicating no apparent immune inflammation was induced at this observation time point. Although the administered hOPCs were present in the brains of fetal goats approximately 21 days after delivery, it remains unclear whether immune rejection occurs over a prolonged period. Therefore, future studies with long‐term observations are required.

In this study, we assessed the levels of inflammatory and neurotrophic factors in each group. We concluded that HI + hOPCs administration significantly reduced the TNF‐α levels compared with the HI + NS group. This suggested that hOPCs may exert additional effects including improvement of the inflammatory microenvironment in addition to building the myelin sheath. Moreover, we determined the concentrations of neurotrophic factors and found that HI decreased the levels of GDNF and BDNF whereas hOPC administration increased these levels. This suggests that hOPCs exert additional therapeutic effects by regulating neurotrophic factors including GDNF and BDNF. Neurotrophic factors (mostly produced by neurons and glial cells) contributed to neuroprotective and pro‐myelinating effects [38]. GDNF, mostly produced by astroglia and oligodendrocytes has been reported to mediate neuroprotective effects, including the prevention of neuronal loss and regulation of blood‐nerve barrier tight junction formation [39, 40]. Additionally, BDNF exhibits neuronal protective and pro‐myelination effects, promoting neuronal growth, differentiation, and survival [41]. Moreover, BDNF enhances OPC proliferation and differentiation by binding to the TrkB neurotrophin receptor [42]. Studies have reported that OPC transplantation in rat models of WMI reduces neuronal apoptosis, enhances endogenous neural stem cell proliferation, and alleviates the loss of neurons and oligodendrocytes [43, 44]. Therefore, we speculate that hOPCs may mediate additional therapeutic effects such as protecting neurons and promoting the survival and myelination of endogenous oligodendrocytes by increasing the levels of GDNF and BDNF.

Despite the potential of this treatment strategy, the safety of hOPC administration must be considered. First, hOPCs were not observed in the liver, spleen, heart, lungs, or kidneys at 21 days after administration indicating a low risk of off‐target effects. Secondly, pluripotent stem cell‐derived OPCs may be mixed with undifferentiated residual cells, and OPCs (being a type of stem cell) can theoretically exhibit tumorigenicity [45]. However, previous studies did not report on tumor formation even 2 years after OPCs administration in experimental animal studies [8]. In our study, transplanted hOPCs barely expressed Ki67, indicating a low capacity for proliferation in vivo. However, similar to immune rejection, tumorigenicity is a critical concern that requires long‐term monitoring. Future studies monitoring proliferation or tumor‐specific markers will help eliminate the potential risk of tumorigenesis.

However, the optimal time window for stem cell administration in patients with PWMI remains unclear. Previous studies have reported that the early administration of umbilical cord blood cells at 12 h after HI reduced WMI [19, 35]. In addition, considering the time required to implement stem cell therapy, 12 h is a relatively suitable time window. Therefore, in the present study, hOPCs were administered 12 h after HI.

This study has several limitations. First, we only included a sham group (placement of an inflatable silicone occluder and nasal catheter without UCO or hOPCs administration) in our preliminary experiment. WMIs and other abnormalities were not observed in the sham group. To ensure comparable gestational age, white matter developmental stage, and intrauterine environment, we used twin siblings of HI fetuses as the control group instead of the sham group. Second, prolonged observation periods including functional recovery after delivery, were not included in our study.

Studies on OPCs for the treatment of PWMI are still in progress, and safety issues such as tumorigenicity and immune rejection after OPCs transplantation need to be continuously monitored. Further studies are required to determine the optimal therapeutic dose, route and duration of drug administration, and recipient selection. Furthermore, transplanted OPCs may be damaged by the inflammatory microenvironment in brains with HI. Therefore, seeking methods to improve the microenvironment after brain injury is important. Most stem cell‐related technologies are still under development. There are several challenges in the application of stem cell technology in clinical practice. First, stem cell technology incurs high research and manufacturing costs, which include building standardized and fully automated cell production methods to scale up stem cell production. Second, stem cell therapy involves a variety of technologies that require strict safety assessments and regulations. In addition, the use of human cells or tissues requires an ethical review and evaluation. Third, stem cell technology requires interdisciplinary cooperation such as the integration of biomedicine, materials science, and computer science, which will provide new perspectives for innovation in stem cell technology. Moreover, the application of new technologies such as Clustered Regularly Interspaced Short Palindromic Repeats (CRISPR) is expected to enhance the therapeutic potential of stem cells.

## 5 Conclusion

PWMI is one of the most serious conditions affecting preterm infants. However, no clinical treatments are currently available for this condition. In this study, we discovered that exogenous hOPCs could differentiate into mature oligodendrocytes in fetal goats and alleviate HI‐induced PWMI. Therefore, hOPC transplantation is a promising strategy for managing PWMI.

## Author Contributions

Y.Y., B.D. and D.M.: Conceived and designed the research; Y.Y., L.S., Y.Z., W.L., X.Q., P.H. and T.R.: Establishment of the animal model; B.D., S.L., J.Y.: Analysis and interpretation of data; Y.Y, B.D. and D.M: Drafting of the manuscript; T.X., Y.Q., Z.L. and D.M.: Critical revision of the manuscript for important intellectual content; S.L., Y.Q., and D.M.: Administrative, technical, or material support.

## Conflicts of Interest

The authors declare no conflicts of interest.

## Supporting information


Data S1.

Data S2.

## Data Availability

The data that support the findings of this study are available from the corresponding author upon reasonable request.
