# Supplementary material for: Oligodendrocyte Progenitor Cell Transplantation Reduces White Matter Injury in a Fetal Goat Model
Source: CNS Neurosci Ther. 2024 Dec 17;30(12):e70178. doi: 10.1111/cns.70178 (PMC11652673; doi:10.1111/cns.70178)
Supplement: Supplementary file 1 — Data S1. [file CNS-30-e70178-s002.docx]

**Supplemental Figures**


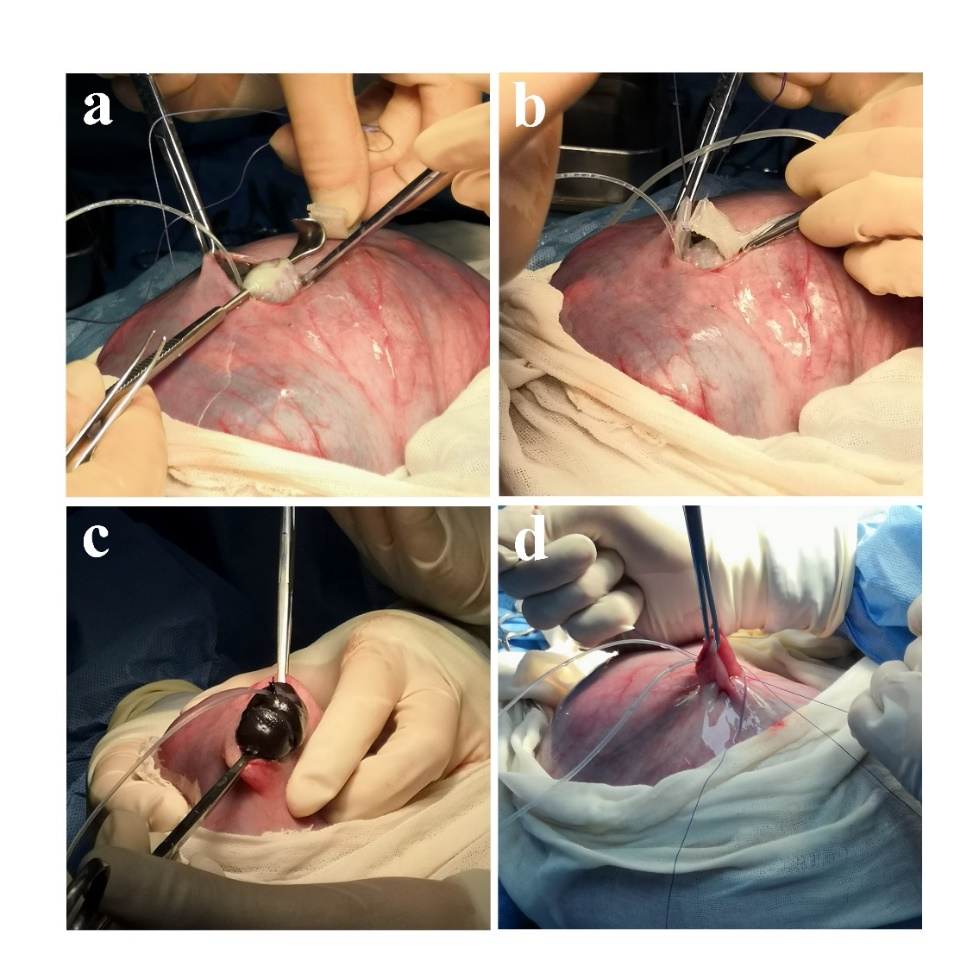


**sFigure 1.** **Main surgical procedures for placing the inflatable silicone occluder and the nasal polyvinyl catheter.**

(a) The uterus of the mother is exposed, and the umbilical cord of the fetus is identified. (b) The umbilical cord is wrapped with an inflatable silicone occluder. (c) A polyvinyl nasal catheter is inserted into one of the nasal cavities. (d) The uterine incision is sutured.


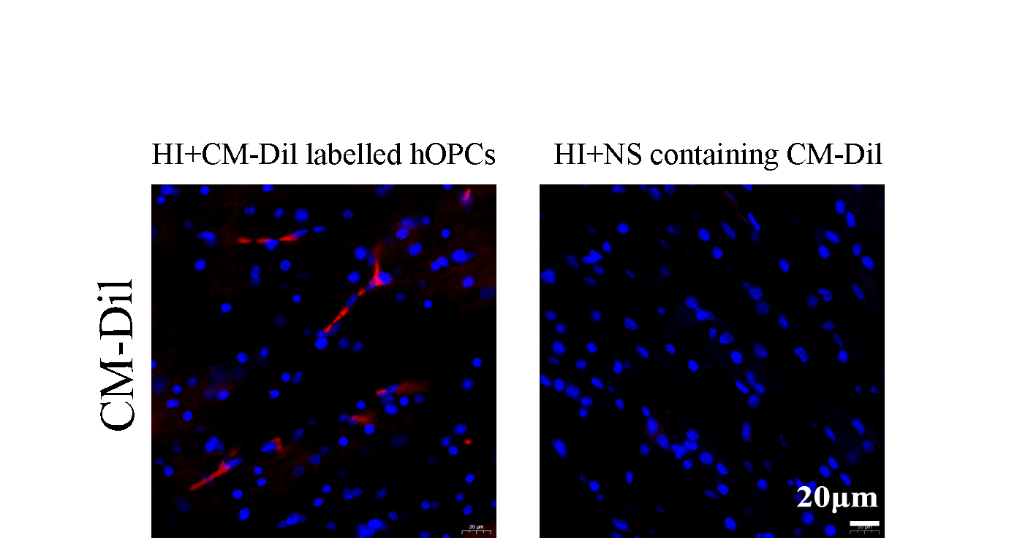


**sFigure 2. Human oligodendrocyte precursor cells (hOPCs) are detected in the transplanted brain.** CM-DiI-labeled hOPCs are detected in the brains of hOPC-treated fetuses. Red fluorescence is not observed in the brains of animals treated with normal saline containing CM-DiI Red dye. The brain tissue was collected 14 days after transplantation. Scale bar=20 μm, n=3 for each group.


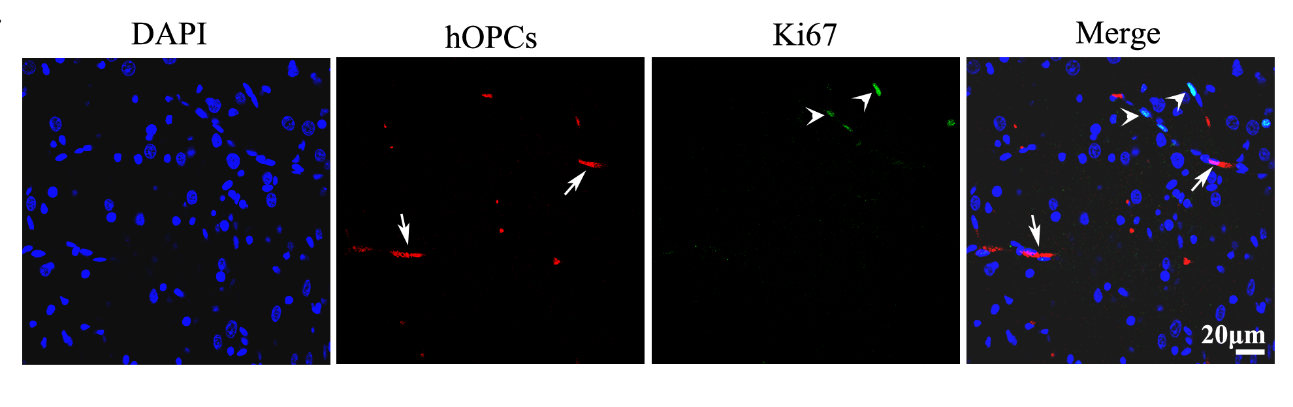


**sFigure 3. Administered human oligodendrocyte precursor cells (hOPCs) have low proliferation ability in the brain.** At 21 days, immunofluorescence staining confirms that the administered hOPCs barely expressed Ki67 (3.9%±3.2%), a marker of proliferating cells. Arrows indicate hOPCs. Arrowheads indicate Ki67-positive cells. Scale bar =20 μm, n=4.


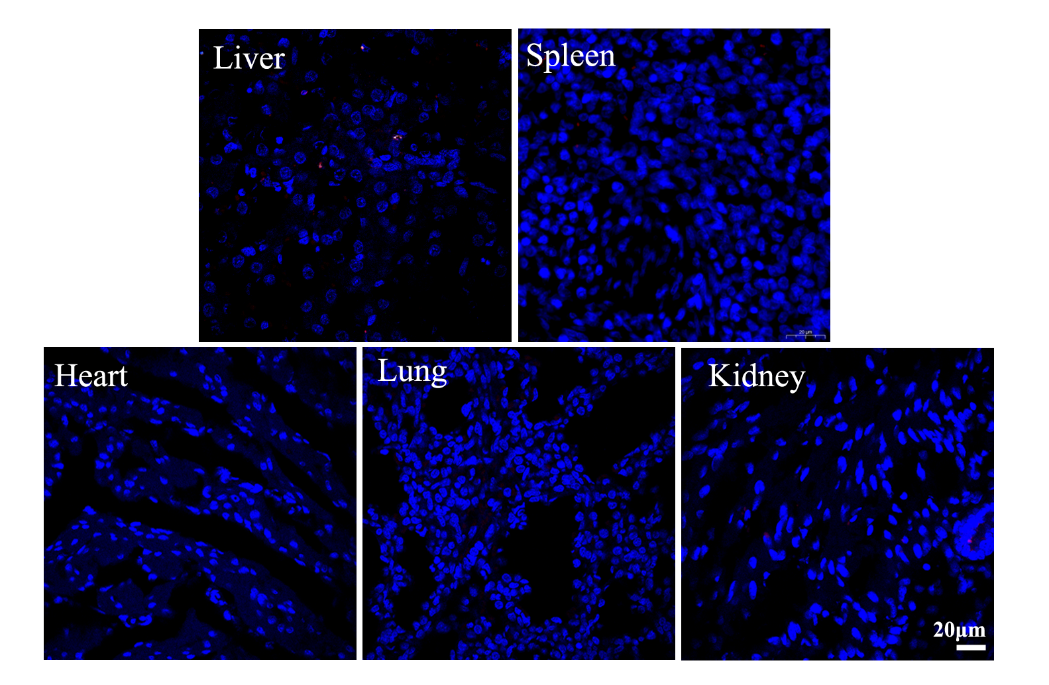


**sFigure 4. Human oligodendrocyte precursor cells (hOPCs) not identified in internal organs at 21 days after administration.** CM-DiI-labeled hOPCs are not detected in the liver, spleen, heart, lungs, or kidneys. Scale bar =20 μm, n=4.
