# Supplementary material for: Oligodendrocyte Progenitor Cell Transplantation Reduces White Matter Injury in a Fetal Goat Model
Source: CNS Neurosci Ther. 2024 Dec 17;30(12):e70178. doi: 10.1111/cns.70178 (PMC11652673; doi:10.1111/cns.70178)

Full unedited blot for Figure 6C

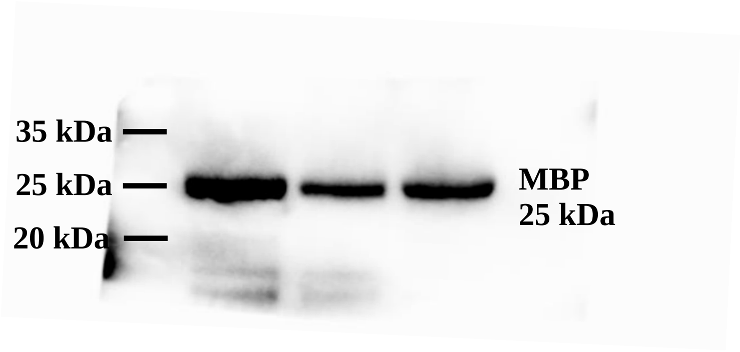

Full unedited blot for Figure 6C

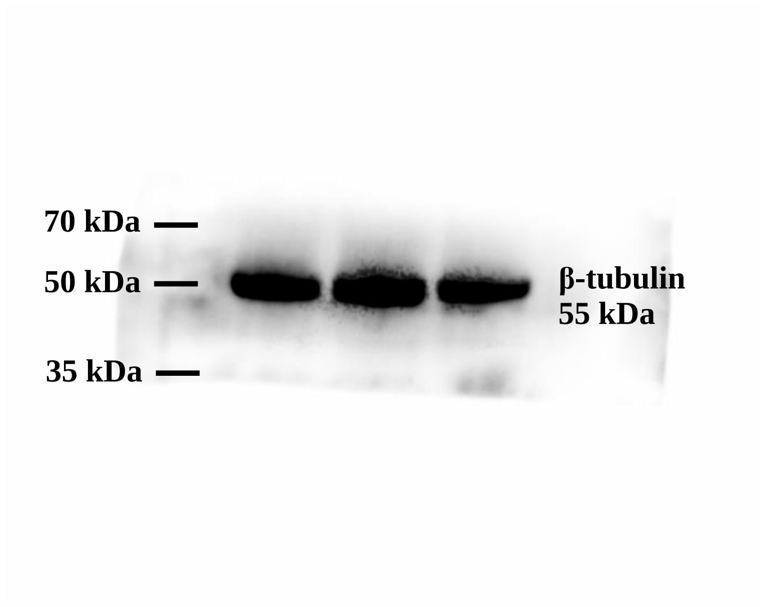

Full unedited blot for Figure 6C

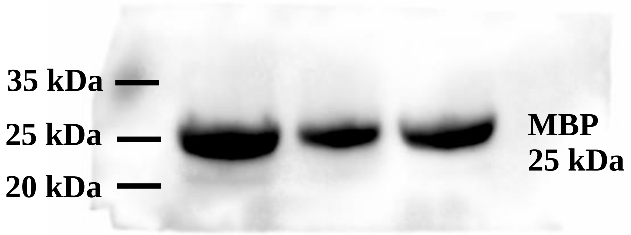

Full unedited blot for Figure 6C

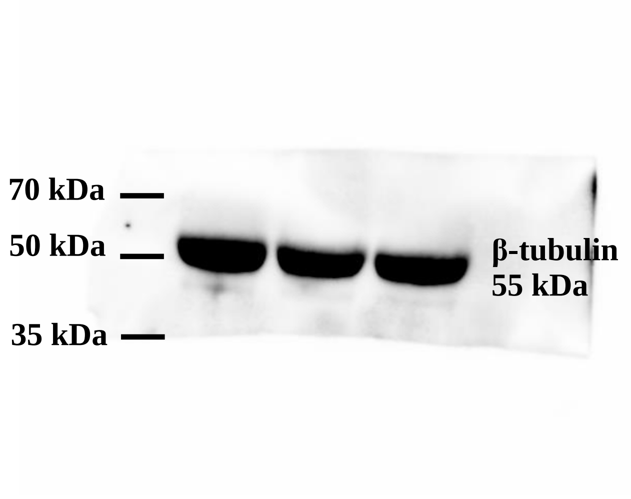

Full unedited blot for Figure 7C

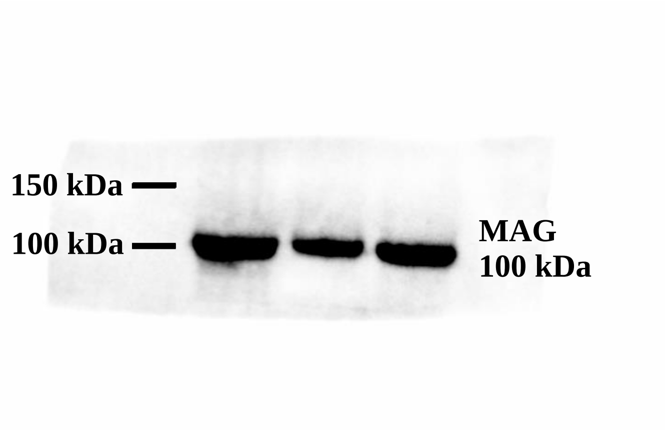

Full unedited blot for Figure 7C

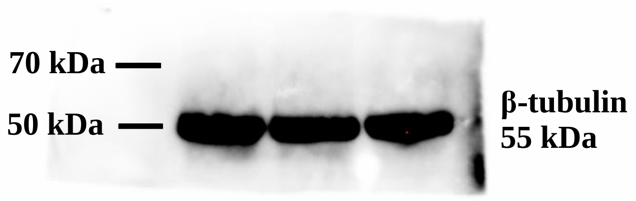

Full unedited blot for Figure 7C

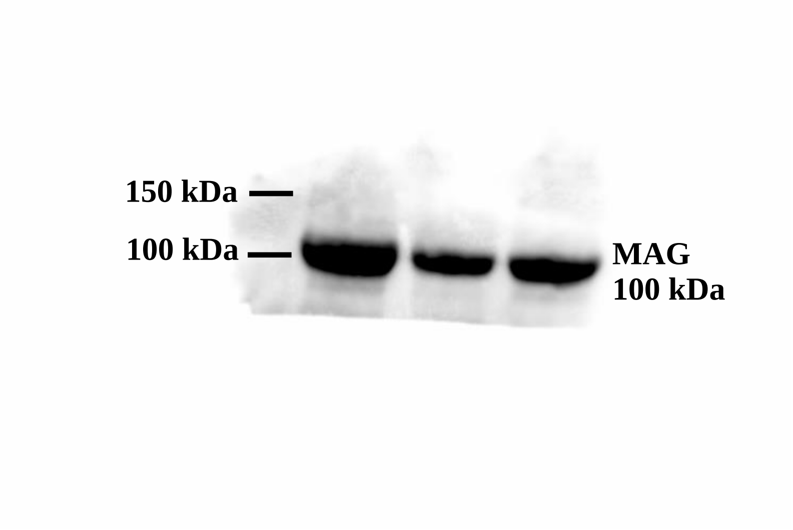

Full unedited blot for Figure 7C

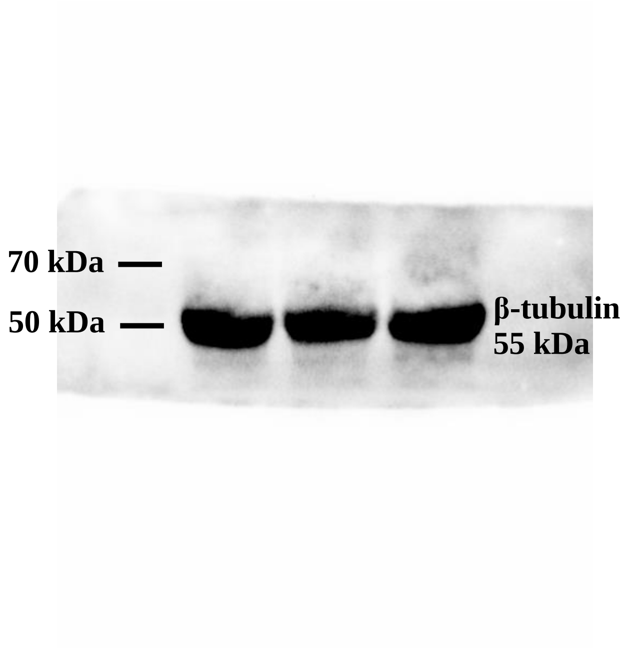

Supplement: Supplementary file 2 — Data S2. [file CNS-30-e70178-s001.pdf]
